# Asia–Pacific consensus statement on integrated 24-hour activity guidelines for the early years

**DOI:** 10.1016/j.lanwpc.2022.100641

**Published:** 2022-11-23

**Authors:** Benny Kai Guo Loo, Anthony Okely, Rachael Taylor, Rachel Novotny, Pujitha Wickramasinghe, Falk Müller-Riemenschneider, Gade Waqa, Aman Pulungan, Satoshi Kusuda, Kok Hian Tan, Asia–Pacific 24-Hour Activity Guidelines for the Early Years Committee

**Affiliations:** a Sport and Exercise Medicine Service, KK Women’s and Children’s Hospital, Singapore; b School of Health and Society, University of Wollongong, Sydney, New South Wales, Australia; c Department of Medicine, University of Otago, Dunedin, Otago, New Zealand; d Department of Human Nutrition, Food and Animal Sciences, University of Hawaiʻi at Mānoa, Honolulu, HI, USA; e Faculty of Medicine, University of Colombo, Colombo, Sri Lanka; f Saw Swee Hock School of Public Health, National University of Singapore, Singapore; g School of Public Health and Primary Care, Fiji National University, Suva, Fiji; h Endocrinology Division, Child Health Department, Faculty of Medicine, Universitas Indonesia – Dr Cipto Mangunkusumo Hospital, Jakarta, Indonesia; i Department of Pediatrics, Kyorin University, Mitaka, Tokyo, Japan; j Department of Maternal Fetal Medicine, KK Women’s and Children’s Hospital, Singapore

**Keywords:** Infant, Toddler, Pre-schooler, Physical activity, Sedentary behaviour, Sleep, Diet

## Abstract

**Background:**

Early childhood is a vital period for development and growth. Promoting beneficial lifestyle behaviours in early childhood can help optimise children's health, development and learning, shape their behaviours in adulthood and offer the best protection against future non-communicable diseases (NCDs). In the Asia–Pacific region, NCDs are significant causes of healthcare burden and mortality. Furthermore, there is also a high prevalence of adverse metabolic risk factors and unhealthy lifestyle behaviours among these children.

**Method:**

Representatives from 19 Asia–Pacific nations and/or jurisdictions developed a consensus statement on integrated 24-hour activity guidelines for the early years using the GRADE-ADOLOPMENT framework.

**Findings:**

These guidelines apply to all infants, toddlers and pre-schoolers below 5 years of age. The guidelines aim to provide a holistic and practical approach to lifestyle activities by framing physical activity, sedentary behaviour and sleep within a 24-hour period. Dietary patterns were included as they play an integral role in metabolic health and energy balance.

**Interpretation:**

Aligned with the World Health Organization's Global Action Plan for the Prevention and Control of NCDs through health promotion interventions in early life, through cultivating healthy lifestyle behaviours in the children's early years, we aim to provide children with the best start in life and reduce the burden of future NCDs in the Asia–Pacific region.

**Funding:**

Funded by Integrated platform for research in advancing metabolic health outcomes of women and children

**Research in context**

**Evidence before this study**

The committee reviewed the evidence for the World Health Organization (WHO) guidelines on physical activity, sedentary behaviour and sleep for children under 5 years of age, multiple national 24-hour movement/activity guidelines for children 5 years and below, and international and regional eating and dietary guidelines. The committee also updated the evidence by searching PubMed, Embase and CENTRAL databases, using the combination of keywords including “infant”, “toddler”, “preschooler” or “pre-schooler”, “physical activity”, “sedentary behaviour”, “sleep”, and “diet” or “dietary” for studies published in English from January 2018 to December 2021.

The evidence showed that physical activity improves motor development, physical fitness, bone and skeletal health, body composition and emotional well-being. Outdoor play was also protective against incident myopia. Excessive screen time is found to be associated with overweight/obesity in children and may adversely affect their emotional and behavioural well-being. Longer duration of screen time may also promote unhealthy eating habits or shorter sleep duration. Having a consistent bedtime and adequate duration of sleep are associated with lower body mass index. Infants with persistent sleep problems may exhibit more emotional symptoms. Breastfeeding and breastmilk may improve the child's motor and cognitive development compared to feeding infant formula, and that parents who practise responsive feeding is also associated with healthier weight gain in their children. Combinations of the activities are associated favourable with body composition, motor and cognitive development, and combining dietary with physical activity interventions has better body mass reduction effects than either intervention alone.

**Added value of this study**

This is the first set of regional guidelines for Asia–Pacific nations to reduce the morbidity and mortality from non-communicable diseases (NCDs) using international evidence and regional data. Aligned with the WHO's Global Action Plan for the Prevention and Control of NCDs (2013–2020), which emphasised that exposure to NCD risk factors starts in early life, these guidelines promote beneficial lifestyle behaviours in early childhood in order to optimise their health in their growing years, shape their behaviours in adulthood and offer the best protection against future NCDs. This is also the first set of activity guidelines for children 5 years and below to include healthy dietary patterns as they have integral roles in determining metabolic health and energy balance. Through the review of regional data, we have also identified a high prevalence of adverse metabolic risk factors and unhealthy behaviours among the children, and the relationship with the local social or cultural factors. Hence, the recommendations in the guidelines are crafted specifically to cater for the children in the Asia–Pacific region.

**Implications of all the available evidence**

The Asia–Pacific region is susceptible to worsening healthcare burden and mortality caused by NCDs as many young children in the region do not display healthy lifestyle behaviours, and this situation is probably worsened by the COVID-19 pandemic. Through the adoption or adaptation of these evidence-based guidelines, Asia–Pacific nations can cultivate healthy lifestyle behaviours in the children's early years using a holistic and practical approach framed within a 24-hour period, so as to provide them the best start in life, cushion the impact of COVID-19 and reduce the burden of future NCDs in the Asia–Pacific region.

## Introduction

The objective of the Asia–Pacific consensus statement on integrated 24-hour activity guidelines for the early years was to provide physicians and healthcare providers with the latest evidence-based recommendations on beneficial lifestyle habits for infants, toddlers and pre-schoolers. Families of these young children are encouraged to adopt a holistic approach towards integrating different types of activity within a 24-hour period – considering physical activity, sedentary behaviour, sleep and dietary pattern.

These guidelines apply to infants, toddlers and pre-schoolers below 5 years of age, including those living with disability, in the Asia–Pacific region, regardless of gender, cultural background, or socio-economic status. Infants, toddlers and pre-schoolers living with chronic medical conditions should consult a health professional for additional guidance.

All participating members are encouraged to adopt or adapt the guidelines for their local population. Following these guidelines is associated with multiple health benefits, including better body composition, cardiovascular and metabolic health, musculoskeletal fitness, mental and bone health, cognitive performance and overall quality of life. Through these recommendations, we aim to support infants, toddlers and pre-schoolers in the Asia–Pacific region achieve these health benefits.

## Background

Non-communicable diseases (NCDs) are significant causes of healthcare burden and mortality in the Asia–Pacific region.^1^ NCDs caused 86% of deaths in the Western Pacific region as compared to 71% worldwide^2^; NCD-related deaths before the age of 60 years was 34% in South East Asia region in contrast to 23% in the rest of the world.^3^ The modifiable lifestyle behavioural risk factors of NCDs include insufficient physical activity and unhealthy diets, which increases a person's metabolic risk factors such as obesity or high blood pressure.^4^

The World Health Organization's (WHO's) Global Action Plan for the Prevention and Control of NCDs (2013–2020) emphasised that exposure to NCD risk factors starts in early life.^5^ Early childhood is also a vital period for both development and growth. Therefore, promoting beneficial lifestyle behaviours in early childhood can help to optimise children's health in their growing years, shape their behaviours in adulthood and offer the best protection against future NCDs.^5^^,^^6^

The Asia–Pacific region has a high prevalence of adverse metabolic risk factors (e.g. obesity), as well as unhealthy lifestyle behaviours among children.^7^, ^8^, ^9^ Furthermore, there are many middle income nations in this region that have been adversely impacted by NCDs.^4^ Social and cultural factors such as suboptimal dietary and physical activity patterns in the Pacific region and non-responsive parental feeding practices, whereby parents do not recognise or respond to hunger and satiety cues in their children, in the South East Asia region have been reported. These factors have contributed to a disproportionate burden of overweight and obesity in the population, especially among children.^10^^,^^11^

There are reports that the physical activity levels of young children in the Asia–Pacific region are low in comparison with their counterparts in the West,^12^^,^^13^ and physical activity levels usually decline with age.^6^ For example, studies have shown that 89% of pre-schoolers in Taiwan and 34% in Japan do not meet national physical activity recommendations,^14^^,^^15^ 65% of pre-schoolers in China and 44% in Australia do not meet the WHO total physical activity recommendation,^16^^,^^17^ and 40% of those in Singapore do not meet the WHO recommendation for moderate-to vigorous-intensity physical activity (moderate-intensity physical activity is equivalent to 4–7 metabolic equivalent of tasks, METs, in children and >7 METs is classified as vigorous-intensity).^18^ On the other hand, screen time behaviour is becoming more prevalent in young children in the Asia–Pacific region. For example, in Malaysia and Australia, almost 30% of pre-schoolers exceeded the recommended daily amount of screen time.^17^^,^^19^ Similarly, 89% of 2 to 5-year-olds in India and 3 to 4-year-olds in China reported more than 1 h of daily screen time.^20^^,^^21^ The mean amount of sedentary behaviour in pre-schoolers in Bangladesh was found to be 7 h per day,^22^ while the median for pre-schoolers in Singapore was 7.8 h per day.^23^

Sleep behaviour has also been shown to affect growth and development in young children. Shorter sleep duration was shown to be associated with higher body mass index in 3-month-olds in Singapore,^24^ whereas sleep disturbances have been associated with emotional and/or behavioural problems in 4–5 year-olds in China.^25^ Sleep deprivation is an important issue in this region, with only an estimated 9% of Japanese 5-year-olds achieving the recommended amount of sleep daily.^26^

Similarly, unhealthy dietary patterns are common in young children in the Asia–Pacific region. For instance, more than a third of 2- to 6-year-olds in Sri Lanka consume excessive amount of sugar,^27^ high sodium intake has been observed in 30 to 60-month-olds in Hong Kong and pre-schoolers in Taiwan,^28^^,^^29^ and pre-schoolers in China do not consume adequate fruits and vegetables.^30^

Concerns have been expressed that these data have been adversely impacted by the COVID-19 pandemic with several reports showing that the amount of physical activity, especially outdoor play,^31^ has been curtailed and the amount of sedentary behaviour, including screen time, has increased in young children.^32^, ^33^, ^34^ The sedentary lifestyle has also resulted in later bedtimes and shorter sleep durations, and possibly more snacking behaviours.^35^^,^^36^ The lack of physical activity may also adversely impacts on the children's mental wellness.^37^

Importantly, it is increasingly apparent that investigation of the relationships between various types of activities (i.e. physical activity, sedentary behaviour and sleep) and young children's health should consider all of the activities together rather than in isolation.^38^ Interventions incorporating different domains (e.g promoting healthy levels of physical activity, sedentary behaviour and sleep) may also achieve more success than those focusing on only a solitary domain.^39^ This modern concept of integrating these activities within a 24-hour period has been introduced in several national guidelines since 2016 and subsequently adopted by WHO in 2019.^40^, ^41^, ^42^, ^43^ However, dietary patterns also play integral roles in determining metabolic health and energy balance.^44^^,^^45^ Therefore, the workgroup has included recommendations on healthy dietary patterns into the guidelines where feasible. Packaging these activities within a 24-hour period emphasises that the whole day matters and provides a practical perspective in managing these activities.

This paper represents a continuation of the Asia–Pacific 24-Hour Activity Guidelines for Children and Adolescents Committee's effort to develop a set of region-specific recommendations for infants, toddlers and pre-schoolers, so as to consolidate the Asia–Pacific nations' efforts in combating NCDs through health promotion.^46^ These recommendations are also timely due to the adverse impacts of COVID-19 pandemic on the lifestyle and/or metabolic health of children. This report outlines the process and outcomes for the development of the “Asia–Pacific Consensus Statement on Integrated 24-Hour Activity Guidelines for the Early Years”.

## Methods

### Timeline and process

**Fig. 1:**
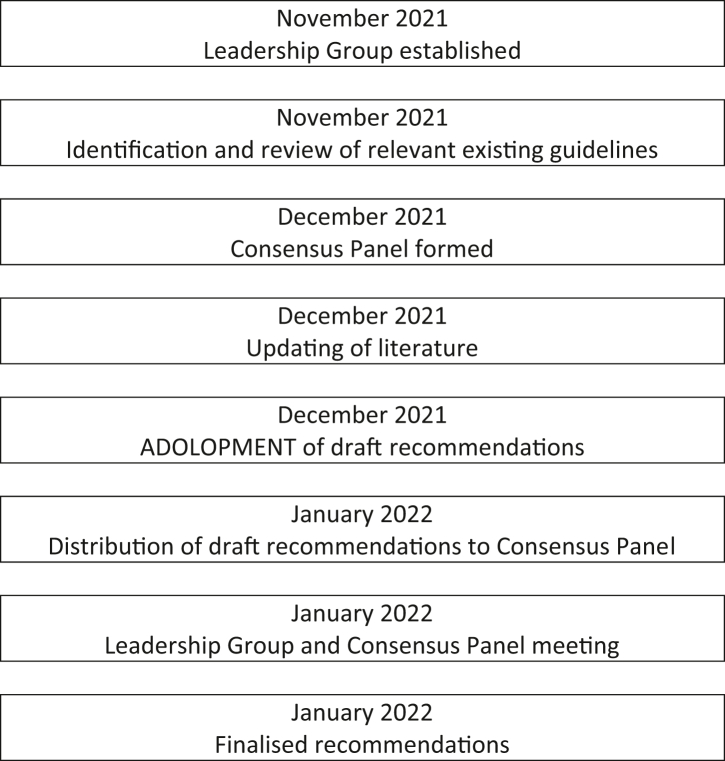
Timeline and processes involved in the development of the Asia–Pacific consensus statement on integrated 24-hour activity guidelines for the early years.

A leadership group, consisting of members with prior experience in local or regional guidelines development or committee work, was formed in November 2021 to guide the overall process. Monthly discussions were held to assess the current situation in the Asia–Pacific region, review the evidence and to draft the recommendations. The consensus panel was formed by December 2021 and the draft recommendations were distributed to the consensus panel in early January 2022. A virtual meeting was held on 22nd January for the leadership group and consensus panel to finalise the recommendations. The timeline and development process are presented in Fig. 1.

### Search strategy and selection criteria

The evidence reviews conducted for the development of the WHO guidelines on physical activity, sedentary behaviour and sleep for children under 5 years of age, Canadian 24-hour movement guidelines for the early years,^40^^,^^47^, ^48^, ^49^ Australian 24-hour movement guidelines for the early years,^41^ South African 24-hour movement guidelines for birth to 5 years,^42^ and the Singapore integrated 24-hour activity guidelines for early childhood were included in the literature review for the development of the Asia–Pacific guidelines.^50^ We also reviewed the evidence used for international and regional eating and dietary guidelines. As the WHO workgroup updated the evidence in December 2017, we conducted an evidence update from January 2018 to December 2021 using PubMed, Embase and CENTRAL databases. The searches included systematic reviews and studies on physical activity, sedentary behaviour, sleep, integration of these three behaviours, and dietary patterns for children under 5 years of age. Only studies published in English language were considered.

### Update of evidence

For physical activity, 11 additional studies were identified – five systematic reviews, two randomized controlled trials and four observational studies. The evidence showed that physical activity improves motor development, physical fitness, bone and skeletal health, body composition, and emotional well-being.^51^, ^52^, ^53^ Outdoor play was also found to have protective effect for risk of incident myopia.^54^

For sedentary behaviour, eight additional studies were identified – two systematic reviews and six observational studies. The evidence showed that excessive screen time is associated with overweight/obesity in children and may adversely affect their emotional and behavioural well-being.^55^^,^^56^ Longer duration of screen time may also promote unhealthy eating habits or shorter sleep duration.^56^^,^^57^

For sleep, eight additional studies were identified – one systematic review, two randomized controlled trials and five observational studies. The evidence showed that a consistent bedtime and having adequate duration of sleep are associated with lower body mass index.^58^^,^^59^ Infants with persistent sleep problems may exhibit more emotional symptoms.^60^

For dietary pattern, nine additional studies were identified – two systematic reviews, one randomized controlled trial and six observational studies. The evidence showed that breastfeeding and breastmilk may improve the child's motor and cognitive development compared to feeding infant formula,^61^ and that parents who practise responsive feeding is also associated with healthier weight gain in their children.^62^

For integrated activities, five additional studies were identified – two systematic reviews, one randomized controlled trial and two observational studies. The evidence suggests that combinations of the activities are associated favourable with body composition, motor and cognitive development.^63^^,^^64^ Combining dietary and physical activity interventions has better body mass reduction effects than either intervention alone.^39^

### Limitations

As only studies published in English language were considered, there is a small possibility that those published in other languages from the Asia–Pacific region may have been omitted. However, it is unlikely that these information would alter the phrasing of the recommendations as there was a wide Asia–Pacific representation on the committee. Another limitation is the higher likelihood of downgrading epidemiological observational evidence when using GRADE.

### Grading of evidence and recommendations

The Grading of Recommendations Assessment, Development and Evaluation (GRADE) method was applied to rate the quality of evidence and strength of recommendations.^65^ The ratings from reference guidelines were also reviewed. The GRADE-ADOLOPMENT framework was employed to provide a structured and transparent methodology for healthcare decisions and recommendations.^66^ This framework included the formulation of the public health question, review of relevant resources, assessment of the evidence and drawing conclusions for healthcare recommendations.

We structured the public health question from the perspective of healthcare providers with the aim to improve the metabolic and general health of infants, toddlers and pre-schoolers in the Asia–Pacific region. The conclusions based on the judgements for all criteria in the assessments showed that they were in favour of the recommendations and the overall evidence was at least moderately certain to support the recommendations. The summary of findings table (for randomized controlled trials) and full framework are included as Table 1 and in the Annex respectively.

**Table 1:** Summary of findings for randomized controlled trials.

Summary of findings: Health effects of holistic lifestyle recommendations for infants, toddlers and preschoolers^a^				
Outcomes	Control group (no. of participants)	Intervention group (no. of participants)	Health effects in Intervention group^b^	Certainty of the evidence (GRADE)
Beneficial effects of physical activity^c^	871	678	• Improved performance in cognitive tasks related to numerical tasks (mean score 35.25; SE: 1.97 vs 22.07; SE 2.09) • Improvement in velocity/agility (boys −2.51s; 95% CI −3.98, −1.05; girls −2.35s; 95% CI −3.71, −0.98) • Improvement in muscular strength (boys 0.66 cm/kg; 95% CI: 0.03,1.28; girls 1.26; 95% CI: 0.03, 1.28) • Improvement in cardiorespiratory fitness (girls 1.19; 95% CI: 0.31, 2.08)	⊕⊕⊕○^d^
Beneficial effects of sleep^e^	116	161	• Earlier bedtime associated with lower body mass index (decrease in z-score 0.02; 95% CI: 0.00, 0.04), intake of added sugars (decrease by 2.51 g; 95% CI: 0.70, 4.32) and sweet food servings (decrease by 0.10 h; 95% CI: 0.00, 0.19) • Longer night-time sleep associated with fewer added sugars (95% CI, −3.79 to −0.35; 0.02) • Napping ≥5×/day at 1-month was significantly associated with decreased odds for rapid weight gain compared to napping <5× (OR = 0.11, 95% CI: 0.02, 0.63)	⊕⊕⊕○^f^
Beneficial effects of dietary pattern^g^	60	59	• Consumed ∼94 kcal or 23% less daily energy from solid fat and added sugar (mean 307.8 kcal; 95% CI: 274.1, 341.5 vs. 401.9; 95% CI: 369.8, 433.9) • Mothers displayed a greater number of authoritative parenting practices when observed post-intervention with their child at a buffet-style meal	⊕⊕○○^h^
Beneficial effects of relationships of activity^i^	209	196^j^	• Lower BMI *z* scores at age 3.5 years (−0.18; 95% CI: −0.37, 0.02) and at age 5 years (0.06; 95% CI: −0.29, 0.16)^k^ • Lower rates of obesity at age 3.5 years (0.80; 95% CI: 0.44, 1.44) and at age 5 years (0.68; 95% CI: 0.28, 1.67)	⊕⊕⊕○^l^

a Only randomized controlled trials are presented.

b Health effects are presented instead of relative or absoluate effects due to wide heterogeneity of measurements used.

c ^67^^,^^68^.

d Downgraded for indirectness.

e ^69^^,^^70^.

f Downgraded for indirectness.

g ^71^.

h Downgraded for risk of bias and indirectness.

i ^64^.

j Only participants in the combination group is presented.

k ^72^.

l Downgraded for imprecision.

### Expert panel voting

The consensus panel consisted of members of the Asia–Pacific 24-Hour Activity Guidelines for Children and Adolescents Committee, Asia Pacific Pediatric Association and Federation of Asia and Oceania Perinatal Societies. The number of representatives increased to 19 Asia–Pacific nations/jurisdictions – Australia, Bangladesh, China, Fiji, Hawaii, Hong Kong, India, Indonesia, Japan, Malaysia, Mongolia, Myanmar, New Zealand, Philippines, Singapore, South Korea, Sri Lanka, Thailand and Vietnam.

The consensus session was conducted on 22nd January 2022 at the Asia Pacific Maternal and Child Health Conference and Integrated Platform for Research in Advancing Metabolic Health Outcomes of Women and Children International Meeting 2022. For the consensus process, each recommendation was presented and revised according to comments from the representatives. This was followed by an online vote of which there were two responses – ‘Agree’ or ‘Disagree’. Each representative was allowed one vote per recommendation and a majority in agreement was considered when there were 70% or more votes for ‘Agree’. All 13 revised recommendations received a majority in agreement.

### Role of funding source

Funding source is not involved in writing of the manuscript or the decision to submit it for publication.

## Consensus statement

### Infants (0–11 months)

#### Physical activity

Be physically active several times a day in different types of activities and within a safe and supervised floor play environment, where more activity is better. Non screen-based, interactive floor-based play is encouraged. For those not yet mobile, this includes at least 30 min of supervised tummy time spread throughout the day.

##### Supporting paragraph

Physical activity confers many health benefits to infants including improvements in motor skill development, psychosocial health, adiposity and cardiometabolic health indicators.^47^ Tummy time is suitable for infants who are not yet mobile and this is performed as awake prone positioning while on a firm surface.^43^^,^^53^ Tummy time can positively affect global development (i.e. improvement in multiple developmental domains),^73^ especially gross motor development,^74^ body mass index and also in preventing development of brachycephaly.^75^^,^^76^ It is safe to start infants on tummy time soon after birth and aim to build up from a few minutes at a time to accumulate at least 30 min spread throughout the day. The infant is encouraged to play and should be supervised by an adult caregiver during tummy time. For infants above 3 months of age, they can work towards accumulating at least 1 h of prone activities spread throughout the day.

#### Sedentary behaviour

Avoid restraining infants for more than 1 h at a time. Any form of screen time, including background screen time, is not recommended for infants. When sitting, reclining, or lying down, caregivers are encouraged to engage infants in singing, reading, storytelling and/or imaginative play.

##### Supporting paragraph

Infants should not be restrained or strapped (e.g. in car seats, strollers or high chairs) for more than 1 h at a time.^43^ Time spent restrained limits the ability of infants to move around freely and play. Any form of screen time is not recommended for infants. This includes background screen time (e.g. television that is turned on) which can continue to draw an infant's attention or distract them. As infants have immature memory and attentional skills, they cannot process and learn from screen time as they do from interactions with caregivers.^77^ Screen time in infants is also unfavourably associated with sleep duration and quality as well as gross motor development.^78^, ^79^, ^80^ When infants are in sedentary positions (i.e. sitting, reclining, or lying down on their backs), they should be engaged in interactive activities with a caregiver for improved cognitive development.

#### Sleep

Ensure infants 0–3 months old have a total of 14–17 h and infants 4–11 months old have 12–16 h of daily sleep, including naps. Caregivers are recommended to place their infants to sleep on their back, in their own sleeping space such as a cot or bassinet, in the same room as their caregivers, to maintain sleep safety.

##### Supporting paragraph

Infants spend the majority of the time sleeping, with newborns sleeping up to 80% of the time.^81^ Benefits of good sleep include good cognitive, physical and social outcomes.^82^, ^83^, ^84^, ^85^ Risks of obesity and sudden infant death syndrome are also reduced with good sleep.^60^^,^^86^ Good infant sleep is an important predictor of maternal health and improves family well-being.^87^^,^^88^ Infants should be provided with a conducive sleep environment to improve sleep duration. Regular bedtime routines can be initiated for infants from 2 to 3 months of age.^89^^,^^90^

It is recommended that infants share a room with the parents or caregiver, but have their own cot, bassinet or bedside co-sleeper (an infant bed that attaches to the side of the adult bed).^91^, ^92^, ^93^ Infants must always be placed on their back when sleeping.^91^^,^^92^^,^^94^ Prone sleeping (i.e. sleeping on their tummy) is not advised.^91^^,^^95^ The infant sleep environment must be free of items that could cover the infant's face, such as comforters, bumper pads and pillows, and thus place their breathing at risk.^91^^,^^94^

#### Dietary patterns

Exclusive breastfeeding is recommended for the first 6 months of life, where feasible. Around 6 months, or when the infant has shown developmental readiness for complementary food, introduce a variety of nutrient-dense and culturally appropriate solid food of various textures and flavours, while continuing breastfeeding. Prepare food with no added salt or sugar.

##### Supporting paragraph

Infants receive nutrition and antibodies from breast milk for growth, development and health, especially during the first 6 months of life.^96^^,^^97^ It is recommended to exclusively breastfeed infants at least from birth to 6 months of age. In populations at risk of vitamin deficiency, supplementation of 400IU of vitamin D per day is recommended for fully and partially breastfed infants.^97^^,^^98^ In situations where mother's own milk is unavailable, infants should be provided with iron-fortified infant formula. Fresh cow milk or plant-based milks should not be offered in place of infant formula.

Caregivers should adhere to food safety and hygiene recommendations when handling infant formula, and if breast milk is expressed and stored.^99^ Freshly expressed milk can be stored at room temperature for 6 h, and in the refrigerator for 3 days at 5–10 °C and for 5 days below 4 °C. Breast milk frozen below −18 °C can also be stored up to six months.^99^ Thawed (i.e. warmed to liquid form) human milk should not be refrozen and neither should it be microwaved. Once offered to the infant, the unused amounts should be discarded after 2 h. For preparation of infant formula, use cooled boiled water and follow the preparation according to the manufacturer's instruction. Specialized infant formula should only be used after consultation with a physician. Toddler milk and self-importation of infant formula from overseas should not be recommended as the infant formula is a regulated food item which undergoes stringent inspection by the food regulatory bodies to meet the standards of safety and nutritional requirements. As energy and nutrient needs exceed what breast milk can provide, infants should begin complementary foods around 6 months of age, according to their developmental readiness.^97^^,^^98^ Infants should be able to sit with support and maintain head control. Infants should also be ready to grasp food items and to bring to their mouths.^97^ Breastfeeding or infant formula should continue until for at least 12 months as the main source of nutrition, along with the complementary feeding.^97^^,^^98^ Night or nocturnal feeds and bottle feeding should be weaned off as it will predispose to dental caries.

A variety of foods across all the main food groups (grains and alternatives, lean proteins and alternatives, dairy, fruits, and vegetables) should be introduced sequentially, with textures of complementary foods suited to the infant's stage of development. It is recommended that iron-containing foods be encouraged from 6 months of age as the first line of protection against iron deficiency in breastfed infants to support neurologic development and immune function.^97^^,^^98^ Examples of iron-containing foods include iron-fortified infant cereals, poultry and meat, plain tofu or legumes. If food options are not available, supplements can be given to infants who are exclusively breastfed. Food should be prepared without added salt as infants are unable to excrete excess sodium due to immature kidneys. Fresh foods are preferred where feasible, instead of commercial prepared infants or processed foods.^97^

Water is not necessary in the first 6 months but when adding complementary food, fluorinated water can be given in small amounts spread across the day. Avoid food and drinks with added sugars to prevent dental caries and learned preference for sugar. Excess intake of sugar-laden foods or drinks is associated with increased risk of overweight or obesity.^97^^,^^98^^,^^100^^,^^101^ If there is a family history of atopy or eczema, it is advisable to introduce egg or peanut one at a time, around 6 months of age when the infant is developmentally ready.^97^^,^^98^ There is no evidence that food allergies can be prevented by delaying the introduction of potentially allergenic food.^97^ Therefore, potential allergenic foods such as egg, wheat, peanuts and dairy products should be introduced when starting the infant on complementary feeding.

Exposing the infant repeatedly to a variety of food from the main food groups can help develop food acceptance and meet the range of nutrient requirements. Caregivers should practice responsive consumption through timely starting and stopping of feeding by learning to recognise an infant's hunger and satiety cues.^62^ Studies have shown that non-responsive feeding practices, such as restrictive, rewarding or pressure feeding may lead to increased risk of childhood obesity. Therefore, it is recommended to provide caregivers with resources on responsive feeding to achieve healthier weight gain in infants.^10^^,^^62^

### Toddlers (1–2 years)

#### Physical activity

Accumulate at least 180 min of a variety of physical activities spread throughout the day within a safe environment; more activity is better. Supervised outdoor active play is essential.

##### Supporting paragraph

Toddlers should participate in a range of physical activities, including locomotor- (e.g. walking, running), object control- (e.g. throwing and catching of ball) and stability-related (e.g. balancing) activities, at all intensities.^47^ Toddlers who engage in at least an hour of moderate-to vigorous-intensity physical activity (i.e. energetic play) each day had significantly better locomotor and object control skills (i.e. using part of body or a device to control an object).^102^ The physical activities should be fun and encourage exploration, and involve movement skills such as crawling, climbing, running, balancing, and playing with balls. Both structured and unstructured play are essential for a toddler's development. These activities can occur in both indoor and outdoor environments.^103^ As active interactions with the child are associated with better developmental skills, reduced risk for obesity, and accumulate physical activity, caregivers should engage actively with the toddler during indoor and outdoor play.^104^^,^^105^ Improving childcare infrastructure with the inclusion of modifiable open spaces can decrease sedentary time and encourage physical activity for toddlers.^106^^,^^107^ Having a minimum of 2 h of outdoor play daily can reduce the risk of incident myopia.^108^ Increased playtime and amount of moderate-to vigorous-intensity physical activity outdoors have positive effects on sleep outcomes in toddlers.^79^

#### Sedentary behaviour

Avoid restraining toddlers for more than 1 h at a time. Sedentary screen time, regardless of the type of device, is not recommended for toddlers younger than 2 years of age. For those between 2 and 3 years old, sedentary screen time should be less than 1 h per day. When sitting, reclining, or lying down, caregivers are encouraged to engage toddlers in singing, reading, storytelling and/or imaginative play.

##### Supporting paragraph

Toddlers should not be restrained or strapped (e.g. in car seats, strollers or high chairs) for more than 1 h at a time.^43^ Time spent restrained limits the ability of toddlers to move around freely and play, and prolonged restraining in a seat or supine position is adversely associated with higher adiposity and poorer motor development.^48^^,^^109^ Children younger than 2 years of age should not engage in screen time as they are not able to learn from it due to their immature memory and attentional skills, and difficulty assimilating the content to their 3-dimensional experience.^77^

For children aged 2 years and older, sedentary screen time refers to the use of any screen device whilst sitting, reclining or lying down.^110^ Screen-based sedentary behaviour is associated with increased adiposity and decreased scores on measures of psychosocial health and cognitive development.^48^ Screen-based sedentary behaviour can negatively impact on motor development, social skills (ability to interact and develop relationships with others effectively), physical activity and sleep outcomes in early childhood.^48^^,^^79^ When toddlers are required to be sedentary, caregivers should engage them in interactive activities such as reading, singing and storytelling where feasible, as these activities are more effective in promoting cognitive and social development.^48^^,^^111^

#### Sleep

Have a daily total of 11–14 h of sleep including regular naps. Keep to a regular bedtime and wake-up time where feasible. Having a bedtime routine or ritual may help the toddler fall asleep.

##### Supporting paragraph

Toddlers with shorter sleep durations are associated with negative health outcomes in later childhood, such as obesogenic eating behaviours, higher blood pressure, poorer temperament and depressive symptoms.^58^^,^^112^, ^113^, ^114^, ^115^ To attain the recommended 11–14 h of sleep, an early bedtime is important. Late bedtime in toddlerhood may affect neurobehavioural development and is associated with attention and aggressiveness problems during school going age.^116^^,^^117^

Develop a bedtime routine and keep to a consistent bedtime where possible,^59^^,^^118^ as these practices promote better quality and duration of sleep in toddlers,^59^^,^^118^ and possibly help to prevent obesity.^119^ Pre-bedtime activities like storytelling or cuddling also help toddlers sleep better and longer.^120^ Provide a dark and quiet environment with comfortable ambient temperature that is conducive for sleep, and avoid screen time at least 30 min before bedtime.^121^ Screens emit blue light that is thought to suppress endogenous melatonin production, which negatively affects sleep duration, sleep latency and bedtimes.^122^^,^^123^ Unfavourable sleeping environment (e.g. crowded or noisy) can also result in shorter sleep durations, longer sleep latency and hence later bedtimes.^124^^,^^125^

#### Dietary patterns

Continue to increase the variety of food, across all key food groups, offered to your toddler. Choose fresh food over highly-processed food, where feasible. Avoid food and drinks with added sugar or high in salt. Introduce healthy family meals and encourage water to drink.

##### Supporting paragraph

Caregivers play an important role in shaping toddlers' feeding habits by deciding the type, timing and how food is consumed.^97^^,^^98^^,^^126^^,^^127^ Establish a healthy home food environment including regular family eating habits with a wide exposure to all major food groups, creating positive atmosphere and minimising distractions (e.g. toys) during meal times, and prepare fresh foods with little or no added salt and sugar. Transition the toddler's meals towards family meals, which recommends that meals need not be prepared separately for the child. This exposes the child to a variety of meal types that is modelled after the parent, and should follow the healthy dietary pattern synchronous with national guidelines.^97^

A toddler's predominant nutrition should no longer be from breast milk or infant formula, and there is no requirement to continue formula milk after 12 months of age.^97^ Continued provision of breast milk, or inclusion of pasteurised full cream milk or fortified unsweetened soy milk in the diet from 12 months of age can provide protein, calcium and vitamin D.^97^^,^^98^ It is not recommended to consume carbonated and non-carbonated sugar-sweetened beverages such as fruit juice, sweetened yoghurt drinks and soft drinks; and caffeinated beverages such as tea, coffee and cola drinks, before 2 years of age.^97^^,^^128^ Instead, plain water should be consumed for hydration needs and the recommended amount of fluid intake is 1 L or more daily.^98^^,^^129^ In keeping with nutrition goals for toddlers, sodium should be limited to 1200 mg per day,^97^ equivalent to half a teaspoon of table salt per day. Commercial foods that may be mid-meal snack options such as crackers, bread and cheese, are also sodium-containing foods that contribute to a toddler's daily sodium intake. As taste preferences for salty foods can be established from a young age, caregivers should refrain from providing toddlers with large amounts of ultra-processed foods, which are usually created by a series of industrial techniques and processes (e.g. carbonated soft drinks, salty packaged snacks, pre-prepared pasta and pizza, and ‘instant’ soups).^130^

Effective responsive feeding practices include forming a structured routine for meal and snack times and having caregivers recognise toddlers’ hunger and satiety cues with appropriate reactions.^126^^,^^127^ Studies have shown that responsive feeding practices are associated with better weight outcomes in toddlers.^127^ Using food to pacify toddlers can lead to overeating and is associated with higher risk of obesity in early childhood.^98^ It is natural for toddlers to have picky eating while they are developing their feeding habits and they should not be forced to eat new foods.^97^^,^^98^ In order to promote familiarity and acceptance, they should be exposed to non-preferred foods frequently and regularly.

### Pre-schoolers (3–4 years)

#### Physical activity

Accumulate at least 180 min of physical activity, of which at least 60 min should include a variety of moderate-to vigorous-intensity activities, spread throughout the day and within a safe environment. Outdoor active play is encouraged.

##### Supporting paragraph

There are numerous health benefits for pre-schoolers when participating in physical activity, particularly when engaging in energetic play at moderate to vigorous intensities (e.g. playing catching or tag at the playground, casual or competitive ball games, and gymnastics or dancing).^47^^,^^51^^,^^131^ Evidence suggests that physical activity has positive effects on adiposity prevention, physical fitness, motor and cognitive development, cardiometabolic and bone health, and psychosocial well-being.^40^^,^^47^^,^^131^ Movement competency in young children is also associated with physical activity participation later in life; hence, it is important for pre-schoolers to engage in a wide range of physical activities incorporating fundamental movement skills and age-appropriate sports in a safe environment.^132^, ^133^, ^134^

Outdoor active play is beneficial for the healthy growth and development of pre-schoolers.^135^, ^136^, ^137^ Spending time outdoors instead of indoors provides more opportunities for energetic play/moderate-to vigorous-intensity physical activity, and thus offer additional health benefits.^137^^,^^138^ Myopia is a major health issue in many East Asian cities within the region due to increasing academic pressure, and daily outdoor play for at least 2 h can help to prevent the onset of myopia.^54^^,^^108^ Outdoor play also confers many opportunities for children's holistic development and learning, as well as opportunities for parent-child bonding.^139^

#### Sedentary behaviour

Limit the total daily amount of sedentary time, such as sitting, reclining, or lying down. Break up extended periods of sedentary time. Recreational sedentary screen time, regardless of the type of device, should be limited to less than 1 h per day.

##### Supporting paragraph

The WHO guidelines on sedentary behaviour in this age group recognise that sedentary time may include time spent engaged in activities (e.g. drawing, colouring, singing) or quiet play (e.g. puzzles, block building), without electronic media.^43^ These activities have cognitive benefits and are important for child development. However, children should not be in sedentary positions for extended periods of time. Regular movement breaks and active play are essential to minimise adverse health effects of staying still too long.^140^

While some forms of screen viewing can be educational in nature, recreational screen time should not exceed 1 h per day; less is better.^43^ Recreational screen-based sedentary behaviour displaces physical activity and has detrimental effects on fitness, adiposity, movement behaviours and sleep in early childhood.^141^^,^^142^ Instead of using screen devices for recreational activities, caregivers are encouraged to engage them in reading, drawing or imaginative play where feasible.^48^^,^^111^ When engaging in recreational screen time, high quality, well-designed programmes are recommended, and should be co-viewed with a caregiver.^77^ It is also important for parents to be good role models and limit their own media viewing, in order to reduce childhood screen time.^143^

#### Sleep

Have a total of 10–13 h of daily sleep, which may include naps. Keep to a regular bedtime and wake-up time where feasible. Having a bedtime routine or ritual may help the pre-schooler fall asleep.

##### Supporting paragraph

Having the recommended hours of sleep has positive associations with health outcomes including physical, psychological and cognitive well-being. Conversely, shorter sleep duration is associated with many negative health effects, such as higher adiposity levels,^144^^,^^145^ more screen time and less physical activity,^146^^,^^147^ higher risk of injuries,^148^^,^^149^ poorer cognitive development and emotional regulation,^150^, ^151^, ^152^ and lower quality of life.^153^ Reduced sleep duration in pre-schoolers is also associated with reduced school readiness.^154^ The total sleep duration includes both naps and nocturnal sleep.

Develop a bedtime routine that includes a wind-down period and avoid screen time at least 30 min before bedtime. Aim to maintain consistent early bedtime and consistent wake-up time daily, across weekdays and weekends. Late or inconsistent bedtime, independently or in interaction with sleep duration, may be associated with childhood and subsequent adolescent obesity.^155^^,^^156^ Provide a dark and quiet environment with comfortable ambient temperature that is conducive to help pre-schoolers sleep better.^121^

#### Dietary patterns

Develop or maintain a healthy dietary pattern through the selection of nutrient-dense food to meet food group needs. Choose fresh food over highly-processed food, where feasible. Avoid food with added sugar or high in salt and choose water over sugar-sweetened beverages. Provide regular meal and snack times in appropriate portions to support growth and development.

##### Supporting paragraph

Caregivers have significant roles in shaping the dietary habits in young children, which can persist into adulthood. Through positive role-modelling and setting a consistent routine for household eating, caregivers can positively influence the dietary quality with coordinated family meals and appetite regulation.^97^^,^^129^^,^^157^ Fresh foods should be highly encouraged over processed foods high in sugar and salt. Avoid adding sugar to food and limit the intake of sugar-sweetened foods, carbonated and non-carbonated beverages with added sugars (including natural sweeteners such as honey, fruit juices and syrups) to 10% or less of total energy consumption can reduce the risk of dental caries and obesity.^97^^,^^100^^,^^101^ It is also not recommended to consume beverages with non-sugar substitutes including sugar alcohol (e.g. sorbitol, xylitol) or intense sweeteners (e.g. aspartame, sucralose) as these substitutes may promote a liking for sweet food and drinks. Avoid adding salt to food and intake of processed foods high in salt (processed deli meats, canned food, store-bought sauces or dressings) to limit sodium intake to 1500 mg per day (less than 1 teaspoon of salt).^97^

Having a nutritious breakfast daily is positively related to nutrient intake and body weight.^129^ Caregivers can also help children to develop self-regulation and autonomy in eating habits by practising structure-based or limit-setting strategies.^97^^,^^157^ These include giving suitable meal portions and avoiding screen time during family meals.^97^^,^^157^ Using of screen devices during mealtimes may lead to poor eating habits such as overeating. It is important to balance the allowance and restriction of feeding as too much of either feeding practice may have negative health effects such as overeating and adiposity.^97^^,^^157^ For instance, giving in to pre-schoolers’ food demands excessively may disrupt their capability to eat based on their hunger and satiety cues. Similarly, pre-schoolers may inadvertently learn to manage negative feelings with food if there is excessive restriction of their food intake.^97^^,^^157^

### All age groups (Birth-4.9 years)

#### Integration

Better health, development and well-being can be achieved by increasing adherence to the physical activity, sedentary behaviour, sleep and dietary pattern guidelines.

##### Supporting paragraph

Physical activity, sedentary behaviour, sleep and dietary patterns are associated with each other in terms of health benefits and time use. In general, the most health benefits can be attained by achieving all the recommendations, which includes more physical activity, less sedentary time, longer sleep duration and healthier dietary patterns.^38^^,^^63^^,^^158^^,^^159^ Evidence suggests that meeting some recommendations can give similar health benefits. For instance, reduction of adiposity and improvement of cognitive development can be achieved by having adequate physical activity and sleep duration, or by having less sedentary time with adequate sleep duration.^38^^,^^43^ Moreover, replacing sedentary behaviour with physical activity is favourably associated with motor and fitness development.^38^^,^^43^ Adopting healthy dietary patterns promotes maintenance of a healthy weight and supports growth and development.^44^^,^^45^ Therefore, achieving various combinations of the recommendations might result in comparable health benefits.

## Conclusion

The Asia–Pacific region is susceptible to worsening healthcare burden and mortality caused by non-communicable diseases (NCDs) as many young children in the region do not display healthy lifestyle behaviours. This situation is probably worsened by the COVID-19 pandemic and management strategies and hence timely lifestyle recommendations are needed. Aligned with the WHO's Global Action Plan for the Prevention and Control of NCDs, promotion of beneficial lifestyle behaviours in early life can optimise the children's health in their growing years and protect them against future NCDs. These guidelines provide the latest evidence-based recommendations for a holistic and practical approach to lifestyle activities by framing these activities within a 24-hour period. Dietary patterns were also included as they are integral to one's metabolic health. We urge all nations in the Asia–Pacific region to adopt or adapt these recommendations as by cultivating healthy lifestyle behaviours in the children's early years, we will then be able to provide them the best start in life, cushion the impact of COVID-19 and reduce the burden of future NCDs in the Asia–Pacific region.

## Summary

### Infants (0–11 months)

#### Physical activity

Be physically active several times a day in different types of activities and within a safe and supervised floor play environment, where more activity is better. Non screen-based, interactive floor-based play is encouraged. For those not yet mobile, this includes at least 30 min of supervised tummy time spread throughout the day.

#### Sedentary behaviour

Avoid restraining infants for more than 1 h at a time. Any form of screen time, including background screen time, is not recommended for infants. When sitting, reclining, or lying down, caregivers are encouraged to engage infants in singing, reading, storytelling and/or imaginative play.

#### Sleep

Ensure infants 0–3 months old have a total of 14–17 h and infants 4–11 months old have 12–16 h of daily sleep, including naps. Caregivers are recommended to place their infants to sleep on their back, in their own sleeping space such as a cot or bassinet, in the same room as their caregivers, to maintain sleep safety.

#### Dietary patterns

Exclusive breastfeeding is recommended for the first 6 months of life, where feasible. Around 6 months, or when the infant has shown developmental readiness for complementary food, introduce a variety of nutrient-dense and culturally appropriate solid food of various textures and flavours, while continuing breastfeeding. Prepare food with no added salt or sugar.

### Toddlers (1–2 years)

#### Physical activity

Accumulate at least 180 min of a variety of physical activities spread throughout the day within a safe environment; more activity is better. Supervised outdoor active play is essential.

#### Sedentary behaviour

Avoid restraining toddlers for more than 1 h at a time. Sedentary screen time, regardless of the type of device, is not recommended for toddlers younger than 2 years of age. For those between 2 and 3 years old, sedentary screen time should be less than 1 h per day. When sitting, reclining, or lying down, caregivers are encouraged to engage toddlers in singing, reading, storytelling and/or imaginative play.

#### Sleep

Have a daily total of 11–14 h of sleep including regular naps. Keep to a regular bedtime and wake-up time where feasible. Having a bedtime routine or ritual may help the toddler fall asleep.

#### Dietary patterns

Continue to increase the variety of food, across all key food groups, offered to your toddler. Choose fresh food over highly-processed food, where feasible. Avoid food and drinks with added sugar or high in salt. Introduce healthy family meals and encourage water to drink.

### Pre-schoolers (3–4 years)

#### Physical activity

Accumulate at least 180 min of physical activity, of which at least 60 min should include a variety of moderate-to vigorous-intensity activities, spread throughout the day and within a safe environment. Outdoor active play is encouraged.

#### Sedentary behaviour

Limit the total daily amount of sedentary time, such as sitting, reclining, or lying down. Break up extended periods of sedentary time. Recreational sedentary screen time, regardless of the type of device, should be limited to less than 1 h per day.

#### Sleep

Have a total of 10–13 h of daily sleep, which may include naps. Keep to a regular bedtime and wake-up time where feasible. Having a bedtime routine or ritual may help the pre-schooler fall asleep.

#### Dietary patterns

Develop or maintain a healthy dietary pattern through the selection of nutrient-dense food to meet food group needs. Choose fresh food over highly-processed food, where feasible. Avoid food with added sugar or high in salt and choose water over sugar-sweetened beverages. Provide regular meal and snack times in appropriate portions to support growth and development.

### All age groups (Birth-4.9 years)

#### Integration

Better health, development and well-being can be achieved by increasing adherence to the physical activity, sedentary behaviour, sleep and dietary pattern guidelines.

## Contributors

All authors contributed to the conceptualisation, literature review, methodology, and review & editing of the manuscript.

BKG Loo provided the original draft of the manuscript.

All authors approved the final version of the manuscript.

Members of the committee contributed to the literature review, consensus process and approval of the revised recommendations.

## Data sharing statement

Not applicable.

## Ethical approval information

Not applicable.

## Declaration of interests

All authors reported no conflict of interest.
