# Supplementary material for: Asia–Pacific consensus statement on integrated 24-hour activity guidelines for the early years
Source: Lancet Reg Health West Pac. 2022 Nov 23;32:100641. doi: 10.1016/j.lanwpc.2022.100641 (PMC9918766; doi:10.1016/j.lanwpc.2022.100641)
Supplement: Supplementary File S1 [file mmc1.pdf]

What are the recommendations to provide a holistic approach towards better metabolic and general health for infants, toddlers and pre-schoolers in the Asia-Pacific region?

QUESTION

Question details

Problem: To improve the metabolic and general health of infants, toddlers and pre-schoolers in the Asia-Pacific region

Option: A set of guidelines that provide holistic recommendations towards good metabolic and general health outcomes

Comparison: Separate guidelines targeting different aspects of metabolic health

Main outcomes:

- Beneficial and detrimental effects of Physical Activity
- Beneficial and detrimental effects of Sedentary Behaviour
- Beneficial and detrimental effects of Sleep
- Beneficial and detrimental effects of Dietary Pattern
- Beneficial and detrimental effects of Relationships of Activities (physical activity, sedentary behaviour, sleep and eating activity)

Setting: Asia-Pacific community

Perspective: Healthcare providers

Background

The Asia-Pacific region has a high prevalence of adverse metabolic risk factors (e.g. undernutrition and obesity), as well as unhealthy lifestyle behaviours among the children. Furthermore, there are many middle-income nations in this region that are adversely impacted by both undernutrition and non-communicable diseases (NCDs), a dual burden. Social or cultural factors such as food and physical activity patterns in the Pacific region and non-responsive parental feeding practices in the South East Asia region have been reported to contribute to both underweight and overweight and obesity in the population, especially in children. Early childhood is also a vital period for development and growth. Therefore, promoting beneficial lifestyle behaviours in early childhood can help to optimise children’s health in the growing years, shape behaviours in adulthood and offer the best health and protection against future NCDs.

ASSESSMENT

Problem

Is the problem a priority?

Research evidence

There are reports that the physical activity level of young children in the Asia-Pacific region is generally low and potentially lower than their counterparts in the West. For example, studies have shown that 89% of pre-schoolers in Taiwan and 34% in Japan did not meet national physical activity recommendations. On the other hand, screen time behaviour is becoming more prevalent in these young children. In Malaysia and Australia, almost 30% of pre-schoolers exceeded recommended daily amount of screen time. Sleep deprivation is certainly an important issue in this region, with an estimated 9% of Japanese 5-year-olds achieved the recommended amount of sleep daily. More Asia Pacific regions are also describing unhealthy dietary patterns in the young children. In Sri Lanka, more than a third of 2 to 6-year-olds consume an excessive amount of sugar.

Desirable effects

How substantial are the desirable anticipated effects?

Research evidence

Type of Outcome	*Study Design* / Author	No. of Studies / Persons	Summary of Findings	Certainty of Evidence
Beneficial Effects of Physical Activity	*Systematic Review*			⊕⊕⊕○
	Pate RR et al. 2019[1]	27 studies	For weight status/adiposity, 12 of 15 studies found negative associations between physical activity and one or more measures of the outcome. For bone health, 10 articles based on 4 studies were identified, and 9 studies showed stronger bone in more active children. For cardiometabolic health, 3 studies were identified and findings were limited and inconsistent. For cognition, 2 systematic reviews were identified and findings were limited. For all four health outcomes, evidence of dose-response relationships and effect modification by demographic factors was very limited.	
	Lee RL et al. 2020[2]	8 studies	The study outcome measures of unstructured play were categorized into three aspects of children's physical health, social skills and emotional wellbeing. All studies reported positive impacts on children's physical activity level, social engagement and emotional wellbeing. We conclude that our review with identified impacts would assist future research directions and policy implementation in this promising field	
	Hewitt L et al. 2020[3]	16 studies (4237 persons)	Tummy time was positively associated with gross motor and total development, a reduction in the BMI-z score, prevention of brachycephaly, and the ability to move while prone, supine, crawling, and rolling. An indeterminate association was found for social and cognitive domains, plagiocephaly, walking, standing, and sitting. No association was found for fine motor development and communication.	

	Veldman SL et al. 2021[4]	39 studies	There was moderate evidence for a positive association between physical activity and motor (n = 11 studies) and cognitive development (n = 10 studies) based on consistent findings from studies having low-to-moderate methodological quality. There was insufficient evidence for an association between physical activity and body composition (n = 15 studies), cardiometabolic health indicators (n = 7 studies), social-emotional development (n = 2 studies) and bone health (n = 2 studies) based on inconsistent findings from studies having weak-to-high methodological quality.	
	Virgara R et al. 2021[5]	9 studies (4458 persons)	There was low-certainty evidence that physical activity interventions may have little to no effect on total daily moderate-to-vigorous physical activity compared to no intervention (MD 1.7 minutes, 95% CI –0.42 to 3.82; P = 0.12; 6 trials; 3042 children). Both trials showed an increase in proportion of session spent in moderate-to-vigorous physical activity (moderate-certainty evidence) from 4% to 7.3% of session time; however, only one trial was statistically significant. There was low-certainty evidence that physical activity interventions may lead to little to no reduction in body mass index (BMI) as a measure of cardiovascular health, compared to no intervention (SMD –0.17, 95% CI –0.44 to 0.10; P = 0.22; 4 trials, 1684 children). Physical activity interventions that were delivered online were more cost-effective than in person. Combined results suggest that staff-and-parent and staff-and-child-based interventions may lead to a small increase in overall daily physical activity and a small reduction or no difference in BMI. Process evaluation was assessed differently by four of the included studies, with two studies reporting improvements in physical activity practices, one reporting high programme satisfaction and one high programme fidelity.	
	Randomised Controlled Trial			
	Mavilidi MF et al. 2018[6]	120 persons	Results showed that children who performed task-relevant integrated physical activity performed better than children in all other conditions. In addition, children who performed physical activity, either integrated or nonintegrated, reported higher scores for enjoyment of the instructional method than the two sedentary learning conditions.	

	Martínez-Vizcaíno V et al. 2020[7]	1434 persons	Improvements in cardiorespiratory fitness were seen in girls (1.19; 95% CI 0.31 to 2.08; p=0.008), but not in boys. Finally, there was an improvement in velocity/agility in both girls (−2.51 s; 95% CI −3.98 to −1.05; p=0.001) and boys (−2.35 s; 95% CI −3.71 to −0.98; p=0.001), and in muscular strength in both girls (0.66; 95% CI 0.03 to 1.28; p=0.038) and boys (1.26; 95% CI 0.03 to 1.28; p<0.001).	
	Cohort Study			
	Senju A et al. 2018[8]	1804 persons	In the gross motor domain, significant difference in questionnaire scores was observed between the “could” and “could-not” groups at 6 months (Hedges’ g, 1.83) and persisted until 3 years (Hedges’ g, 0.33). Significant differences were also observed in the communication, fine motor, problem solving, and personal–social domains at 6 months (Hedges’ g, 0.20–0.58) and persisted until 1, 2, 2, and 1.5 years, respectively (Hedges’ g, 0.21–0.25).	
	Barnett LM et al. 2019[9]	178 to 259 persons	Maternal physical activity optimism (4 months; β = 2.43), home physical activity equipment (9 months; β = 0.82), time outdoors – middle (9 months; β = 2.50) and highest tertile (9 months; β = 2.86), time free to move about - highest tertile (19 months; β = 2.41), time with older children - middle (19 months; β = 3.15) and highest tertile (3.5 years; β = 3.00) were predictive of better locomotor scores. Mothers’ own physical activity (9 months; β = − 0.01) and time active with mum – highest tertile (3.5 years; β = − 3.73) were negatively associated with locomotor skill. Time with older children - highest (4 months; β = 2.27) and middle tertile (19 months; β = 2.97), time free to move about – middle (19 months; β = 2.55) and highest tertile (19 months; β = 2.47), and more home equipment (9 months; β = 0.83); (3.5 years; β = 0.17) were predictive of better object control skills. Maternal physical activity knowledge (3.5 years; β = − 3.05) was negatively associated with object control skill.	
	Lingham G et al. 2021[10]	303 persons	Spending more time outdoors during childhood was associated with reduced risk of myopia in young adulthood (multivariable odds ratio [OR] 0.82, 95% confidence interval [CI] 0.69, 0.98). Spending more time outdoors in later adolescence and young adulthood was associated with reduced risk of late-onset myopia (≥ 15 years of age, multivariable OR 0.79, 95% CI 0.64, 0.98). Spending more time outdoors in both childhood and adolescence was associated with less myopia in young adulthood.	

	Johnson W et al. 2021[11]	4666 persons	Effect modification was observed in boys but not girls and, among boys, was stronger for % fat than BMI. In a fully adjusted model for boys, the association between infant weight *Z*-score gain and adolescent % fat was 1.883 (1.444, 2.322) if MVPA < 60 min/day and 1.305 (0.920, 1.689) if MVPA ≥ 60 min/day; the difference between these two estimates being −0.578 (−1.070, −0.087). Similarly, % fat was 2.981 (1.596, 4.367) units higher among boys who demonstrated rapid infant weight gain (+0.67 to +1.34 *Z*-score) compared to normal weight gain (−0.67 to +0.67 *Z*-scores), but having MVPA ≥ 60 min/day reduced this effect size by −2.259 (−3.989, −0.535) units.	
Beneficial Effects of Sleep	*Randomised Controlled Trial*			⊕⊕⊕○
	Simon SL et al. 2019[12]	151 persons	Bedtime and sleep duration were not significantly different between treatment groups from baseline to posttreatment. After adjusting for baseline sleep, earlier bedtime was associated with lower BMIz (95% CI, 0.00-0.04; .03), intake of added sugars (95% CI, 0.70-4.32; .007), and sweet/dessert food servings (95% CI, 0.00-0.19; .04) at posttreatment. Longer night-time sleep duration was associated with fewer added sugars at posttreatment, adjusting for baseline sleep (95% CI, −3.79 to −0.35; .02).	
	Petrov ME et al. 2021[13]	126 persons	By 6 months, 35.7% (n = 45) of infants experienced RWG, and by 36 months 42.3% (n = 41) of toddlers were OW. Napping ≥5x/day at 1-month was significantly associated with decreased odds for RWG compared to napping <5x (OR = 0.11, 95%CI:0.02, 0.63). Each 1-hour increase in nocturnal vs diurnal sleep was associated with greater odds of incident OW at 36 mos (OR = 1.51, 95%CI:1.13, 2.03).	
	Cohort Study			
	Goetz AR et al. 2019[14]	270 persons	HBW-Normal had the longest and Overweight had the shortest mean 24 hr sleep duration across all time points with NBW-Normal falling in-between the two groups. Compared with Overweight, HBW-Normal exhibited longer 24 hr sleep duration at age 6 months with this group difference maintained over infancy and toddlerhood. No group difference was found for NBW-Normal.	
	Systematic Review			

Beneficial Effects of Eating Activity	Spill MK et al. 2019[15]	27 studies	Moderate evidence from randomized controlled trials suggests that providing responsive feeding guidance to teach mothers to recognize and respond appropriately to children's hunger and satiety cues can lead to “normal” weight gain and/or “normal” weight status in children aged ≤2 y compared with children whose mothers did not receive responsive feeding guidance. Moderate evidence from longitudinal cohort studies indicates an association between maternal feeding practices and the child's weight status and/or weight gain, but the direction of effect has not been adequately studied. Restrictive feeding practices are associated with increased weight gain and higher weight status, and pressuring feeding practices are associated with decreased weight gain and lower weight status. Evidence suggests that a mother's feeding practices are related to concerns about her child's body weight.	⊕⊕○○
	Suchdev PS et al. 2020[16]	29 studies (33,147 persons)	Home fortification with MNP, compared with no intervention or placebo, reduced the risk of anaemia in infants and young children by 18% (RR 0.82, 95% CI 0.76 to 0.90; 16 studies; 9927 children; moderate-certainty evidence) and iron deficiency by 53% (RR 0.47, 95% CI 0.39 to 0.56; 7 studies; 1634 children; high-certainty evidence). Children receiving MNP had higher haemoglobin concentrations (MD 2.74 g/L, 95% CI 1.95 to 3.53; 20 studies; 10,509 children; low-certainty evidence) and higher iron status (MD 12.93 µg/L, 95% CI 7.41 to 18.45; 7 studies; 2612 children; moderate-certainty evidence) at follow-up compared with children receiving the control intervention. We did not find an effect on weight-for-age (MD 0.02, 95% CI −0.03 to 0.07; 10 studies; 9287 children; moderate-certainty evidence). Few studies reported morbidity outcomes (three to five studies each outcome) and definitions varied, but MNP did not increase diarrhoea, upper respiratory infection, malaria, or all-cause morbidity.	
	Randomised Controlled Trial			

	Fisher JO et al. 2019[17]	119 persons	At post-intervention, FFF children consumed ~ 94 kcal or 23% less daily energy from SoFAS than children in the control group, adjusting for baseline levels (307.8 (95%CI = 274.1, 341.5) kcal vs. 401.9 (95%CI = 369.8, 433.9) kcal, FFF vs. control; $p < 0.001$). FFF mothers also displayed a greater number of authoritative parenting practices when observed post-intervention with their child at a buffet-style meal (Wilcoxon z = − 2.54, $p = 0.012$). Neither child total daily energy intake nor BMI z-scores differed between groups post-intervention.	
	Cohort Study			
	Closa-Monasterolo R et al. 2018[18]	179 persons	Ca intake at 6 years was positively correlated with LS BMD at 7 years (R = 0.205, p = 0.030). A Ca increase of 100 mg/day explained 19.4% (p = 0.011) of the LS BMD z-score variation, modifying it by 0.089 (0.021, 0.157) units. Children with Ca PA >95% at 5 and 6 or from 4 to 6 years of age showed higher BMD z-scores at the LS and WB levels than children with Ca PA <95% (p < 0.001 and p < 0.05 for LS and WB BMD, respectively). Ca PA >95% maintained over 2 years explained 26.3% of the LS BMD z-score variation (p < 0.001), increasing it by 0.669 (0.202, 1.137). PA >95% maintained over 3 years explained 24.9% of the LS BMD z-score variation, increasing it by 0.773 (0.282, 1.264). The effects of Ca adequacy on WB BMD were similar. Children with PA >95% over 2 years had an Odds ratio of 13.84 and 12 for osteopenia at the LS and WB levels, respectively (p = 0.001).	
	Pang WW et al. 2020[19]	491 persons	Compared to infants fed formula only, those who were bottle-fed breast milk demonstrated significantly better cognitive performance on both the Bayley Scales of Infant and Toddler Development (Third Edition) at 2 years [adjusted mean difference (95% CI) 1.36 (0.32, 2.40)], and on the Kaufman Brief Intelligence Test (Second Edition) at 4.5 years [7.59 (1.20, 13.99)]. Children bottle-fed breast milk also demonstrated better gross motor skills at 2 years than those fed formula [1.60 (0.09, 3.10)]. Among infants fully fed breast milk, those fed directly at the breast scored higher on several memory tasks compared to children bottle-fed breast milk, including the deferred imitation task at 6 months [0.67 (0.02, 1.32)] and relational binding tasks at 6 [0.41 (0.07, 0.74)], 41 [0.67 (0.04, 1.29)] and 54 [0.12 (0.01, 0.22)] months.	

	Carter SA et al. 2020[20]	2997 persons	Infant milk feeding was significantly associated with lumbar spine BMD ($b = -0.028$; 95% CI, − 0.055; − 0.000; p value, 0.047) in males. On average, males who consumed breastmilk alternatives in infancy had lower lumbar spine BMD measurements than those who were fed only breastmilk. These associations remained significant in fully adjusted models. There were no significant associations between infant milk feeding and bone health for females.	
	Lin Q et al. 2021[21]	221 persons	Overall, 84 (38%) children had a steady growth trajectory from birth to 4 years, while the other 137 (62%) children had an early infancy rapid growth trajectory, particularly in the first three months. Compared to children with steady growth, children with early infancy rapid growth had a significantly higher body mass index, waist circumference, and subcutaneous fat. Moreover, weight change trajectory and three eating behaviors (i.e. food responsiveness, satiety responsiveness and food fussiness), not only had independent effects, but also combined (synergistic) effects on the majority of adiposity measures.	
Beneficial Effects of Relationships of Activity	*Systematic Review*			⊕⊕⊕○
	Brown T et al. 2019[22]	153 studies	There is moderate-certainty evidence from 16 RCTs (n = 6261) that diet combined with physical activity interventions, compared with control, reduced BMI (mean difference (MD) −0.07 kg/m2, 95% confidence interval (CI) −0.14 to −0.01), and had a similar effect (11 RCTs, n = 5536) on zBMI (MD −0.11, 95% CI −0.21 to 0.01). Neither diet (moderate-certainty evidence) nor physical activity interventions alone (high-certainty evidence) compared with control reduced BMI (physical activity alone: MD −0.22 kg/m2, 95% CI −0.44 to 0.01) or zBMI (diet alone: MD −0.14, 95% CI −0.32 to 0.04; physical activity alone: MD 0.01, 95% CI −0.10 to 0.13) in children aged 0-5 years.	
	Janssen X et al. 2020[23]	31 studies	Results indicate that screen time is associated with poorer sleep outcomes in infants, toddlers and preschoolers. Meta-analysis confirmed these unfavorable associations in infants and toddlers but not preschoolers. For movement behaviors results were mixed, though physical activity and outdoor play in particular were favorably associated with most sleep outcomes in toddlers and preschoolers.	
	Randomised Controlled Trial			

	Taylor RW et al. 2018[24]	802 persons	Retention was 77% at age 3.5 y and 69% at age 5 y. Children in the FAB group had significantly higher BMI *z* scores than did Controls at age 5 y (adjusted difference: 0.25; 95% CI: 0.04, 0.47) but not at age 3.5 y (0.15; 95% CI: −0.04, 0.34). Children who received the Sleep intervention (Sleep and Combination groups) had significantly lower BMI *z* scores at age 3.5 y (−0.24; 95% CI: −0.38, −0.10) and at age 5 y (−0.23; 95% CI: −0.38, −0.07) than children who did not (Control and FAB groups).	
	Cross-Sectional Study			
	Chia MY et al. 2020[25]	2384 persons	Parent-reported data showed that 12.6% *met none* while 9.6% of preschool children *met all* the WHO guidelines. 70.7%, 56.9% and 26.5%, of preschool children respectively, achieved the sleep, physical activity and screen media use guidelines within a 24-h period. 40.5% *met two* guidelines while 37.4% *met one* guideline. Significant differences were detected in the health-related quality of life among preschool children who *met all*, *none*, or *met 1–2* of the WHO guidelines (i.e. total health score: 82.9 ± 12.4 vs. 76.4 ± 15.1 vs. 78.6 ± 14.5%, $p < 0.05$; $\eta 2 = 0.008–0.11$). Our results show that the health-related quality of life of preschool children increased with the number of WHO guidelines accomplished.	
	Kuzik N et al. 2020[26]	95 persons	Children accumulated 11.1 hours of sleep, 6.1 hours of stationary time, 5.1 hours of light-intensity physical activity (LPA), and 1.8 hours of moderate- to vigorous-intensity physical activity (MVPA) per day. Movement behaviour compositions were significantly associated with physical (i.e., locomotor skills, object motor skills, and total motor skills) and cognitive (i.e., working memory and vocabulary) development (R2 range: 0.11–0.18). In relation to other movement behaviours in the composition, MVPA was positively associated with most physical development outcomes; while stationary time had mixed findings for cognitive development outcomes (i.e., mainly positive associations in linear regressions but non-significant in substitution models). Most associations for LPA and sleep were non-significant.	

Additional considerations

Type of Outcome	Author/Organisation	Type of Paper	Name of Paper
Physical Activity	World Health Organization. 2019	Guidelines	Guidelines on Physical Activity, Sedentary Behaviour and Sleep for Children under 5 Years of Age
	World Health Organization. 2020	Guidelines	WHO guidelines on physical activity and sedentary behaviour
	Health Promotion Board, Singapore. 2013	Guidelines	National Physical Activity Guidelines-Children and Youth Aged up to 18 Years
Sedentary Behaviour	American Academy of Pediatrics. 2013	Policy	Children, adolescents, and the media
Sleep	Paruthi S et al. 2016	Consensus	Recommended Amount of Sleep for Pediatric Populations: A Consensus Statement of the American Academy of Sleep Medicine
	Hirshkowitz M et al. 2015	Consensus	National Sleep Foundation's updated sleep duration recommendations: final report
	American Academy of Pediatrics. 2016	Policy	SIDS and Other Sleep-Related Infant Deaths: Updated 2016 Recommendations for a Safe Infant Sleeping Environment
Eating Activity	World Health Organization. 2020	Guidelines	Healthy Diet
	Health Promotion Board, Singapore. 2020	Guidelines	Early Childhood Nutrition
	World Health Organization. 2019	Guidelines	Essential nutrition actions: mainstreaming nutrition through the life-course
	Phillips JA et al. 2021	Guidelines	Dietary Guidelines for Americans, 2020-2025
	World Health Organization. 2015	Guidelines	Guideline: Sugars Intake for Adults and Children
	Academy of Medicine Singapore. 2019	Consensus	Consensus statement: prevention of allergy in at-risk infants
	Pérez-Escamilla R et al. 2017	Consensus	Feeding guidelines for infants and young toddlers: a responsive parenting approach

	American Heart Association. 2017	Statement	Added Sugars and Cardiovascular Disease Risk in Children: A Scientific Statement from the American Heart Association
	Wellington Ministry of Health. 2015	Guidelines	Food and Nutrition Guidelines for Healthy Children and Young People (Aged 2–18 years): A background paper. Partial Revision ed.
Integrated Activities	Tremblay MS et al. 2017	Guidelines	Canadian 24-Hour Movement Guidelines for the Early Years (0-4 years): An Integration of Physical Activity, Sedentary Behaviour, and Sleep
	Okely AD et al. 2017	Guidelines	A collaborative approach to adopting/adapting guidelines - The Australian 24-Hour Movement Guidelines for the early years (Birth to 5 years): an integration of physical activity, sedentary behavior, and sleep
	Draper CE et al. 2020	Guidelines	The South African 24-Hour Movement Guidelines for Birth to 5 Years: An Integration of Physical Activity, Sitting Behavior, Screen Time, and Sleep

Undesirable effects

How substantial are the undesirable anticipated effects?

Research evidence

Type of Outcome	*Study Design* / Author	No. of Studies / Persons	Summary of Findings	Certainty of Evidence
Detrimental Effects of Sedentary Behaviour	*Systematic Review*			⊕⊕○○
	Li C et al. 2020[27]	80 studies	Strong evidence of the meta-analysis suggested that excessive screen time was associated with overweight/obesity and shorter sleep duration among toddlers and preschoolers. Excessive screen use was associated with various health indicators in physical, behavioral, and psychosocial aspects. Better-quality research on newer media devices, on various kinds of contents in young children, and on dose–response relationships between excessive screen use and health indicators are needed to update recommendations of screen use.	
	Paulus FW et al. 2021[28]	87 studies	Manifold studies describe in young children's utilization data the pervasive nature of digital exposure and impressive usage times and availability. They confirm adverse influences of electronic media use (television, video games) on children's emotional and behavioral problems and well-being, e. g. on physical activity, sleep and obesity. In general, a positive effect in sense of knowledge transfer of age could be found for high quality educational media, however predominantly the impact of media use at younger age was negative.	
	Cohort Study			
	Simonato I et al. 2018[29]	1985 persons	Every 1 h 13 m increase in daily televiewing was prospectively associated with a 8.2% increased risk of unhealthy eating habits (unstandardized $b = 0.05$; 95% CI, 0.02 to 0.07), 10.1% decrease in eating breakfast on weekdays (unstandardized $b = -0.06$; 95% CI, −0.09 to −0.04), 13.3% increase in BMI (unstandardized $b = 0.38$; 95% CI, 0.26 to 0.50), 4.7% decrease in student engagement (unstandardized $b = -0.07$; 95% CI, −0.14 to −0.004), and 5.8% increase in concurrent screen time (unstandardized $b = 0.06$; 95% CI, 0.02 to 0.11). Post hoc simulations of noncompliance with AAP recommendations support their implementation.	

	Aishworiya R et al. 2019[30]	387 persons	The average amount of TV viewing at 12 months was 2.0 h/day (SD 1.9). TV viewing in hours per day was a significant exposure variable for composite IQ (ß = − 1.55; 95% CI: − 2.81 to − 0.28) and verbal IQ (ß = − 1.77; 95% CI: − 3.22 to − 0.32) at 4.5 years. Our path analysis demonstrated that lower maternal education and worse maternal mood (standardized ß = − 0.27 and 0.14, respectively, p < 0.01 for both variables) were both risk factors for more media exposure. This path analysis also showed that maternal mood and infant TV strongly mediated the relationship between maternal education and child cognition, with an exceptional model fit (CFI > 0.99, AIC 15249.82, RMSEA < 0.001).	
	Carson V et al. 2019[31]	251 persons	Across the study, screen time was negatively associated with express (b = − 0.068, 95%CI: -0.114, − 0.023) and comply (b = − 0.056; 95%CI: -0.094, − 0.018) scores and positively associated with disrupt scores (b = 0.004; 95% CI: 0.001, 0.006). Findings were similar for television/videos but less consistent for video/computer games. No associations were observed for physical activity. Screen time significantly tracked at moderate-high levels (β1 = 0.63; 95% CI: 0.45, 0.81), while all other behaviors tracked at moderate levels (β1 = 0.35–0.49; p < 0.01) over the three time-points.	
	Padmapriya N et al. 2019[32]	956 persons	Among boys and girls combined, screen-viewing was positively associated with sum of skinfold thicknesses, but not with BMI or BP. Sex-specific analyses showed significant associations with both BMI and sum of skinfold thicknesses in boys, but not in girls. Screen-viewing was not associated with BP in boys or girls. The increases in mean (95% CI) BMI per hour increase in daily total, television and handheld-devices screen-viewing among boys were 0.12 (0.03, 0.21), 0.18 (0.06, 0.30) and 0.11 (−0.07, 0.29) kg/m2, respectively. The corresponding increases in mean sum of skinfold thicknesses were 0.68 (0.29, 1.07), 0.79 (0.26, 1.32) and 1.18 (0.38, 1.99) mm.	

	Chen B et al. 2020[33]	552 persons	Total screen viewing time at age 2–3 years had a significant negative association with sleep (p=0·008), light physical activity (p<0·0001), and MVPA (p<0·0001) in relation to sedentary behaviour at age 5·5 years. Compared with children who spent 1 h or less per day screen viewing at age 2–3 years, children who screen viewed for 3 h or more per day at 2–3 years engaged in more sedentary behaviour (439·8 mins per day [≤1 h screen viewing time] vs 480·0 mins per day [≥3 h screen viewing time]), and less light physical activity (384·6 vs 356·2 mins per day), and MVPA (76·2 vs 63·4 mins per day) at age 5·5 years. No significant differences in time spent sleeping were observed between the groups (539·5 vs 540·4 mins per day). Similar trends were observed for television viewing and handheld device viewing.	
	Padmapriya N et al. 2021[34]	307 persons	Greater total screen-viewing time and handheld device times were associated with higher superficial and deep subcutaneous adipose tissue volumes, but not with visceral adipose tissue volumes. Interactions with child sex were found, with significant associations with superficial and deep subcutaneous and visceral adipose tissue volumes in boys, but not in girls. Among boys, the increases in mean (95% CI) superficial and deep subcutaneous and visceral adipose tissue volumes were 24.3 (9.9, 38.7), 17.6 (7.4, 27.8), and 7.8 (2.1, 13.6) mL per hour increase in daily total screen-viewing time, respectively. Ethnicity-specific analyses showed associations of total screen-viewing time with abdominal adiposity only in Malay children. Television viewing time was not associated with abdominal adiposity.	
Detrimental Effects of Sleep	*Systematic Review*			⊕⊕○○
	Deng X et al. 2021[35]	33 studies (57848 persons)	Overall analyses revealed statistically significant associations of short (adjusted RR = 1.57, 95% CI: 1.36 to 1.81, P < 0.001) and long sleep duration (0.83, 0.75 to 0.93, 0.001) with obesity. Short sleep duration was also associated with significant changes in body mass index z-score (mean difference = −0.06; 95% CI: −0.09 to −0.04; P < 0.001). By contrast, long sleep duration was identified as a protective factor for childhood obesity. In dose–response analyses, short sleep duration was significantly associated with obesity in toddlers (1–2 years) (adjusted RR = 1.20, 95% CI: 1.07 to 1.34, P = 0.001), preschool-aged (3–5 years) children (1.58, 1.36 to 1.83, <0.001), and school-aged (6–13 years) children (1.82, 1.51 to 2.21, <0.001). In subgroup analyses, geographic region, sleep duration assessment, age, and follow-up interval were possible sources of heterogeneity.	
	Randomised Controlled Trial			

	Covington L et al. 2021[36]	207 persons	Toddlers from households with higher poverty ratios had more inconsistent sleep onset times. Toddlers with more inconsistent sleep onset times had higher BMI *z*-scores across all timepoints, even when accounting for physical activity and diet quality. Sleep onset consistency indirectly explained the association between household poverty and BMI *z*-score.	
	Cohort Study			
	Sparano S et al. 2019[37]	7974 persons	Children reporting shorter sleep duration at T0 had significantly higher BP values (P for trend < 0.001) compared to those who slept more. Prospective analyses showed that shorter sleep duration at baseline predicted, over the 2-year follow-up, higher increases in systolic blood pressure and diastolic blood pressure, after adjustment for age, sex, country of origin, BMI z-score, parental education, physical activity, screen time, and T0 value of the examined outcome variables (P for trend < 0.001).	
	Cook F et al. 2020[38]	1460 persons	283 (19.4%) infants had persistent severe sleep problems, 817 (56.0%) had moderate/fluctuating sleep problems and 360 (24.7%) infants were settled. Infants with persistent severe sleep problems were more likely to report emotional symptoms at age 4 (adjusted odds ratio (AOR)=2.70, 95% CI 1.21 to 6.05, p=0.02), and meet diagnostic criteria for an emotional disorder at age 10 (AOR=2.37, 95% CI 1.05 to 5.36, p=0.04). Infants with persistent severe sleep problems also had elevated symptoms of separation anxiety (AOR=2.44, 95% CI 1.35 to 4.41, p<0.01), fear of physical injury (AOR=2.14, 95% CI 1.09 to 4.18, p=0.03) and overall elevated anxiety (AOR=2.20, 95% CI 1.13 to 4.29, p=0.02) at age 10.	
	Sivertsen B et al. 2021[39]	35075 persons	After accounting for previous internalizing problems, short sleep duration (≤10 hr) and frequent (≥3) nightly awakenings at 1.5 years predicted the development of depressive symptoms at 8 years of age (adjusted OR = 1.28; 95% confidence interval [CI] 1.08–1.51, and adjusted OR = 1.27, 95% CI 1.08–1.50, respectively). Also, internalizing problems at 1.5 years predicted onset of later short sleep duration (adjusted OR = 1.83, 95% CI 1.32–2.54) after accounting for early sleep problems.	

	Reynaud E et al. 2021[40]	1021 persons	Five distinct sleep-duration trajectories were identified. At age 5–6 years, probability of hyperactivity-inattention problems was increased for boys belonging to the "short sleep" and "medium-low sleep" duration trajectory and girls belonging to the "changing sleep" duration trajectory as compared with children with the medium-high sleep duration trajectory (boys: OR 2.69 [95% CI 1.18–6.16], p = .03 and 1.95 [1.25–3.03], p = .003, and girls: OR 2.79 [1.09– 7.17], p = .03). No associations were observed with the other SDQ subscales.	
Detrimental Effects of Eating Activity	*Cohort Study*			⊕⊕○○
	Quah PL et al. 2019[41]	767 persons	Association between SSB intake (100 ml/d increments and tertile categories) and adiposity measures (BMI standard deviation scores (sd units), sum of skinfolds (SSF)) and overweight/obesity status were examined using multivariable linear and Poisson regression models, respectively. After adjusting for confounders and additionally for energy intake, SSB intake at age 18 months were not significantly associated with later adiposity measures and overweight/obesity outcomes. In contrast, at age 5 years, SSB intake when modelled as 100 ml/d increments were associated with higher BMI by 0·09 (95 % CI 0·02, 0·16) sd units, higher SSF thickness by 0·68 (95 % CI 0·06, 1·44) mm and increased risk of overweight/obesity by 1·2 (95 % CI 1·07, 1·23) times at age 6 years. Trends were consistent with SSB intake modelled as categorical tertiles.	

	Vandyousefi S et al. 2021[42]	1035 persons	Adequate BF (≥6 months), adequate EBF duration (≥6 months), and SSB and 100% fruit juice intake in the first year were independently associated with child obesity at ages 2–5 years (all $p < 0.05$). Compared with children with adequate EBF and no intake of SSB or 100% fruit juice, those with adequate EBF and intake of 100% fruit juice and/or SSBs had a four- to fivefold higher odds of obesity (aOR 4.2, 95% CI:1.6, 11.2 for 100% fruit juice; aOR 4.5, 95% CI:1.4, 8.5 for fruit juice or SSBs; and aOR 4.7, 95% CI:1.4, 15 for SSBs; all $p < 0.01$), while those with inadequate EBF (<6 months) and intake of 100% fruit juice and/or SSBs had a six- to 12-fold higher odds of obesity (aOR 6.4, 95% CI:2.4, 17.2 for fruit juice; aOR 6.6, 95% CI:2.7, 14.8 for fruit juice or SSBs; and aOR 12.2, 95% CI:4.3, 25 for SSBs; all $p < 0.001$). Compared with children with adequate BF and no intake of SSB or 100% fruit juice, those with adequate BF and intake of 100% fruit juice and/or SSBs had a threefold higher odds of obesity (aOR 3.1, 95% CI:1.1, 7.3 for fruit juice; aOR 3.3, 95% CI:1.3, 8.3 for fruit juice or SSBs; and aOR 3.4, 95% CI:1.3, 8.5 for SSBs; all $p < 0.05$), while those with inadequate BF (<6 months) and intake of 100% fruit juice and/or SSB were associated with five- to tenfold higher odds of obesity (aOR 4.8, 95% CI:2.3, 12.2 for fruit juice; aOR 6.0, 95% CI:2.5, 12.8 for fruit juice or SSBs; aOR 9.5, 95% CI:3.7, 15.1 for SSBs; all $p < 0.05$).	

Certainty of the evidence

What is the overall certainty of the evidence of effects?

Research evidence

Type of Outcome	Author	Study Design	No. of Studies / Persons	Certainty of Evidence
Beneficial Effects of Physical Activity	Pate RR et al. 2019	Systematic Review	27 studies	⊕⊕⊕○
	Lee RL et al. 2020		8 studies	
	Hewitt L et al. 2020		16 studies (4237 persons)	
	Veldman SL et al. 2021		39 studies	
	Mavilidi MF et al. 2018	Randomised Controlled Trial	120 persons	
	Martínez-Vizcaíno V et al. 2020		1434 persons	
	Senju A et al. 2018	Cohort Study	1804 persons	
	Barnett LM et al. 2019		178 to 259 persons	
	Lingham G et al. 2021		303 persons	
	Johnson W et al. 2021		4666 persons	
Beneficial Effects of Sleep	Simon SL et al. 2019	Randomised Controlled Trial	151 persons	⊕⊕⊕○
	Petrov ME et al. 2021		126 persons	
	Goetz AR et al. 2019	Cohort Study	270 persons	
Beneficial Effects of Eating Activity	Spill MK et al. 2019	Systematic Review	27 studies	⊕⊕○○
	Suchdev PS et al. 2020		29 studies (33,147 persons)	
	Fisher JO et al. 2019	Randomised Controlled Trial	119 persons	
	Closa-Monasterolo R et al. 2018	Cohort Study	179 persons	
	Pang WW et al. 2020		491 persons	
	Carter SA et al. 2020		2997 persons	
	Lin Q et al. 2021		221 persons	

Beneficial Effects of Relationships of Activity	Brown T et al. 2019	Systematic Review	153 studies	⊕⊕⊕○
	Janssen X et al. 2020		31 studies	
	Taylor RW et al. 2018	Randomised Controlled Trial	802 persons	
	Chia MY et al. 2020	Cross-Sectional Study	2384 persons	
	Kuzik N et al. 2020		95 persons	

Type of Outcome	Author	Study Design	No. of Studies / Persons	Certainty of Evidence
Detrimental Effects of Sedentary Behaviour	Li C et al. 2020	Systematic Review	80 studies	⊕⊕○○
	Paulus FW et al. 2021		87 studies	
	Simonato I et al. 2018	Cohort Study	1985 persons	
	Aishworiya R et al. 2019		387 persons	
	Carson V et al. 2019		251 persons	
	Padmapriya N et al. 2019		956 persons	
	Chen B et al. 2020		552 persons	
	Padmapriya N et al. 2021		307 persons	
Detrimental Effects of Sleep	Deng X et al. 2021	Systematic Review	33 studies (57848 persons)	⊕⊕○○
	Covington L et al. 2021	Randomised Controlled Trial	207 persons	
	Sparano S et al. 2019	Cohort Study	7974 persons	
	Cook F et al. 2020		1460 persons	
	Sivertsen B et al. 2021		35075 persons	
	Reynaud E et al. 2021		1021 persons	
Detrimental Effects of Eating Activity	Quah PL et al. 2019	Cohort Study	767 persons	⊕⊕○○
	Vandyousefi S et al. 2021		1035 persons	

Values

Is there important uncertainty about, or variability in, how much people value the main outcomes?

Research evidence

There are existing national physical activity guidelines and active research in both physical activity and sedentary behaviour of children and adolescents in many countries in the Asia-Pacific region.

Balance of effects

Does the balance between desirable and undesirable effects favour the option or the comparison?

Research evidence

There are existing national physical activity guidelines and active research in both physical activity and sedentary behaviour of children and adolescents in many countries in the Asia-Pacific region.

Resources required

How large are the resource requirements (costs)?

Research evidence

The public will benefit from educational or infographic aids for easy understanding and reference.
Healthcare providers will benefit from scientific reports and educational lectures or workshops to improve skills in delivering the recommendations.
Improvement in metabolic and general health will contribute to considerable savings in healthcare expenditure over time.

Certainty of evidence of required resources

What is the certainty of the evidence of resource requirements (costs)?

Research evidence

Improvement in metabolic and general health will contribute to considerable savings in healthcare expenditure over time.
This is assumed to be similar to current campaigns against NCDs in each country.

Cost-effectiveness

Does the cost-effectiveness of the option favour the option or the comparison?

Research evidence

The investment in the public education of these recommendations will contribute to considerable savings in healthcare expenditure over time.
This is assumed to be similar to current campaigns against NCDs in each country.

Equity

What would be the impact on health equity?

Research evidence

These recommendations are available to all children and adolescents in the Asia-Pacific region.
These recommendations can be delivered from primary care, tertiary care and related organisations.

Acceptability

Is the option acceptable to key stakeholders?

Research evidence

The option is likely acceptable to key stakeholders as it is similar to their current campaigns against NCDs.

Feasibility

Is the option feasible to implement?

Research evidence

The option is likely feasible to implement as this approach is similar to their current campaigns against NCDs.

CONCLUSIONS

Summary of judgements

Problem	Don't know	Varies		No	Probably No	Probably Yes	**Yes**
Desirable effects	Don't know	Varies		Trivial	Small	**Moderate**	Large
Undesirable effects	Don't know	Varies		Large	Moderate	**Small**	Trivial
Certainty of the evidence	No included studies			Very low	Low	**Moderate**	High
Values				Important uncertainty or variability	Possibly important uncertainty or variability	**Probably no important uncertainty or variability**	No important uncertainty or variability
Balance of effects	Don't know	Varies	Favours the comparison	Probably favours the comparison	Does not favour either the option or the comparison	**Probably favours the option**	Favours the option

Resources required	– Don't know	– Varies	– Large costs	– Moderate costs	– Negligible costs or savings	**Moderate savings**	– Large savings
Certainty of evidence of required resources	– No included studies			– Very low	– Low	**Moderate**	– High
Cost-effectiveness	– Don't know	– Varies	– Favours the comparison	– Probably favours the comparison	– Does not favour either the option or the comparison	**Probably favours the option**	– Favours the option
Equity	– Don't know	– Varies	– Reduced	– Probably reduced	– Probably no impact	**Probably increased**	– Increased
Acceptability	– Don't know	– Varies		– No	– Probably No	**Probably Yes**	– Yes
Feasibility	– Don't know	– Varies		– No	– Probably No	**Probably Yes**	– Yes

Type of recommendation

Recommendation

<u>1. Infants (0 – 11 months)</u>

1.1 Physical Activity: Be physically active several times a day in different types of activities and within a safe and supervised floor play environment, where more activity is better. Non screen-based, interactive floor-based play is encouraged. For those not yet mobile, this includes at least 30 minutes of supervised tummy time spread throughout the day.

1.2 Sedentary Behaviour: Avoid restraining infants for more than one hour at a time. Any form of screen time, including background screen time, is not recommended for infants. When sitting, reclining, or lying down, caregivers are encouraged to engage infants in singing, reading, storytelling and/or imaginative play.

1.3 Sleep: Ensure infants 0-3 months old have a total of 14-17 hours and infants 4-11 months old have 12-16 hours of daily sleep, including naps. Caregivers are recommended to place their infants to sleep on their back, in their own sleeping space such as a cot or bassinet, in the same room as their caregivers, to maintain sleep safety.

1.4 Dietary Patterns: Exclusive breastfeeding is recommended for the first 6 months of life, where feasible. Around 6 months, or when the infant has shown developmental readiness for complementary food, introduce a variety of nutrient-dense and culturally appropriate solid food of various textures and flavours, while continuing breastfeeding. Prepare food with no added salt or sugar.

2. Toddlers (1 – 2 years)

2.1 Physical Activity: Accumulate at least 180 minutes of a variety of physical activities spread throughout the day within a safe environment; more activity is better. Supervised outdoor active play is essential.

2.2 Sedentary Behaviour: Avoid restraining toddlers for more than one hour at a time. Sedentary screen time, regardless of the type of device, is not recommended for toddlers younger than 2 years of age. For those between 2 to 3 years old, sedentary screen time should be less than one hour per day. When sitting, reclining, or lying down, caregivers are encouraged to engage toddlers in singing, reading, storytelling and/or imaginative play.

2.3 Sleep: Have a daily total of 11-14 hours of sleep including regular naps. Keep to a regular bedtime and wake-up time where feasible. Having a bedtime routine or ritual may help the toddler fall asleep.

2.4 Dietary Patterns: Continue to increase the variety of food, across all key food groups, offered to your toddler. Choose fresh food over highly-processed food, where feasible. Avoid food and drinks with added sugar or high in salt. Introduce healthy family meals and encourage water to drink.

<u>3. Pre-schoolers (3 – 4 years)</u>
3.1 Physical Activity: Accumulate at least 180 minutes of physical activity, of which at least 60 minutes should include a variety of moderate- to vigorous-intensity activities, spread throughout the day and within a safe environment. Outdoor active play is encouraged.

3.2 Sedentary Behaviour: Limit the total daily amount of sedentary time, such as sitting, reclining, or lying down. Break up extended periods of sedentary time. Recreational sedentary screen time, regardless of the type of device, should be limited to less than one hour per day.

3.3 Sleep: Have a total of 10-13 hours of daily sleep, which may include naps. Keep to a regular bedtime and wake-up time where feasible. Having a bedtime routine or ritual may help the pre-schooler fall asleep.

3.4 Dietary Patterns: Develop or maintain a healthy dietary pattern through the selection of nutrient-dense food to meet food group needs. Choose fresh food over highly-processed food, where feasible. Avoid food with added sugar or high in salt and choose water over sugar-sweetened beverages. Provide regular meal and snack times in appropriate portions to support growth and development.

<u>4. All Groups (Birth – 4.9 years)</u>
4.1 Integration: Better health, development and well-being can be achieved by increasing adherence to the physical activity, sedentary behaviour, sleep and dietary pattern guidelines.

Justification

We recommend these guidelines to encourage all infants, toddlers and pre-schoolers in the Asia-Pacific region to adopt a holistic approach towards integrating all types of activity within a daily 24-hour period. These activities (physical activity, sedentary behaviour, sleep and eating activity) are closely interrelated in terms of health benefits and time expenditure. It is equally important to understand the significance of each type of activity and aim to achieve all the recommendations consistently within 24 hours for the best health outcomes.

Detailed justification

- ☒ Problem
- ☒ Desirable effects
- ☒ Undesirable effects
- ☒ Certainty of the evidence
- ☐ Values
- ☐ Balance of effects
- ☐ Resources required
- ☐ Certainty of evidence of required resources
- ☒ Cost-effectiveness
- ☐ Equity
- ☐ Acceptability
- ☐ Feasibility

Problem

Metabolic morbidities and NCDs in children are increasing globally and straining both healthcare resources and expenditure. Lifestyle interventions are vital to improving the metabolic and general health of infants, toddlers and pre-schoolers to combat future NCDs and to cultivate good lifelong habits.

Desirable effects

The evidence is at least moderately certain to recommend the integration of regular physical activity, minimal sedentary behaviour, adequate sleep and good dietary patterns towards good metabolic and general health in infants, toddlers and pre-schoolers.

Undesirable effects

Necessary precautions should be taken when engaging in physical activities and during sleep.

Certainty of the evidence

The review of the evidence consisted of many systematic reviews with large number of studies or participants and the overall evidence is at least moderately certain to support the recommendations.

Cost-effectiveness

Promotion of healthy lifestyle behaviours in early childhood is likely to reduce overall healthcare expenditure in the future.

References

1. Pate RR, Hillman CH, Janz KF, Katzmarzyk PT, Powell KE, Torres A, et al. Physical Activity and Health in Children Younger than 6 Years: A Systematic Review. Med Sci Sports Exerc. 2019;51(6):1282-91.
2. Lee RLT, Lane SJ, Tang ACY, Leung C, Louie LHT, Browne G, et al. Effects of an Unstructured Free Play and Mindfulness Intervention on Wellbeing in Kindergarten Students. Int J Environ Res Public Health. 2020;17(15).
3. Hewitt L, Kerr E, Stanley RM, Okely AD. Tummy Time and Infant Health Outcomes: A Systematic Review. Pediatrics. 2020;145(6).
4. Veldman SLC, Chin APMJM, Altenburg TM. Physical activity and prospective associations with indicators of health and development in children aged <5 years: a systematic review. Int J Behav Nutr Phys Act. 2021;18(1):6.
5. Virgara R, Phillips A, Lewis LK, Baldock K, Wolfenden L, Ferguson T, et al. Interventions in outside-school hours childcare settings for promoting physical activity amongst schoolchildren aged 4 to 12 years. Cochrane Database Syst Rev. 2021;9:CD013380.
6. Mavilidi MF, Okely A, Chandler P, Louise Domazet S, Paas F. Immediate and delayed effects of integrating physical activity into preschool children's learning of numeracy skills. J Exp Child Psychol. 2018;166:502-19.
7. Martinez-Vizcaino V, Pozuelo-Carrascosa DP, Garcia-Prieto JC, Cavero-Redondo I, Solera-Martinez M, Garrido-Miguel M, et al. Effectiveness of a school-based physical activity intervention on adiposity, fitness and blood pressure: MOVI-KIDS study. Br J Sports Med. 2020;54(5):279-85.
8. Senju A, Shimono M, Tsuji M, Suga R, Shibata E, Fujino Y, et al. Inability of infants to push up in the prone position and subsequent development. Pediatr Int. 2018;60(9):811-9.
9. Barnett LM, Hnatiuk JA, Salmon J, Hesketh KD. Modifiable factors which predict children's gross motor competence: a prospective cohort study. Int J Behav Nutr Phys Act. 2019;16(1):129.
10. Lingham G, Yazar S, Lucas RM, Milne E, Hewitt AW, Hammond CJ, et al. Time spent outdoors in childhood is associated with reduced risk of myopia as an adult. Sci Rep. 2021;11(1):6337.
11. Johnson W, Norris T, De Freitas R, Pearson N, Hamer M, Costa S. Is the positive relationship of infant weight gain with adolescent adiposity attenuated by moderate-to-vigorous physical activity in childhood? Evidence from the Millennium Cohort Study. Int J Obes (Lond). 2021;45(1):84-94.
12. Simon SL, Goetz AR, Meier M, Brinton J, Zion C, Stark LJ. Sleep duration and bedtime in preschool-age children with obesity: Relation to BMI and diet following a weight management intervention. Pediatr Obes. 2019;14(11):e12555.
13. Petrov ME, Whisner CM, McCormick D, Todd M, Reyna L, Reifsnider E. Sleep-wake patterns in newborns are associated with infant rapid weight gain and incident adiposity in toddlerhood. Pediatr Obes. 2021;16(3):e12726.
14. Goetz AR, Beebe DW, Peugh JL, Mara CA, Lanphear BP, Braun JM, et al. Longer sleep duration during infancy and toddlerhood predicts weight normalization among high birth weight infants. Sleep. 2019;42(2).
15. Spill MK, Callahan EH, Shapiro MJ, Spahn JM, Wong YP, Benjamin-Neelon SE, et al. Caregiver feeding practices and child weight outcomes: a systematic review. Am J Clin Nutr. 2019;109(Suppl_7):990S-1002S.

16. Suchdev PS, Jefferds MED, Ota E, da Silva Lopes K, De-Regil LM. Home fortification of foods with multiple micronutrient powders for health and nutrition in children under two years of age. Cochrane Database Syst Rev. 2020;2:CD008959.
17. Fisher JO, Serrano EL, Foster GD, Hart CN, Davey A, Bruton YP, et al. Title: efficacy of a food parenting intervention for mothers with low income to reduce preschooler's solid fat and added sugar intakes: a randomized controlled trial. Int J Behav Nutr Phys Act. 2019;16(1):6.
18. Closa-Monasterolo R, Zaragoza-Jordana M, Ferre N, Luque V, Grote V, Koletzko B, et al. Adequate calcium intake during long periods improves bone mineral density in healthy children. Data from the Childhood Obesity Project. Clin Nutr. 2018;37(3):890-6.
19. Pang WW, Tan PT, Cai S, Fok D, Chua MC, Lim SB, et al. Nutrients or nursing? Understanding how breast milk feeding affects child cognition. Eur J Nutr. 2020;59(2):609-19.
20. Carter SA, Parsons CM, Robinson SM, Harvey NC, Ward KA, Cooper C, et al. Infant milk feeding and bone health in later life: findings from the Hertfordshire cohort study. Osteoporos Int. 2020;31(4):709-14.
21. Lin Q, Jiang Y, Wang G, Sun W, Dong S, Deng Y, et al. Combined effects of weight change trajectories and eating behaviors on childhood adiposity status: A birth cohort study. Appetite. 2021;162:105174.
22. Brown T, Moore TH, Hooper L, Gao Y, Zayegh A, Ijaz S, et al. Interventions for preventing obesity in children. Cochrane Database Syst Rev. 2019;7:CD001871.
23. Janssen X, Martin A, Hughes AR, Hill CM, Kotronoulas G, Hesketh KR. Associations of screen time, sedentary time and physical activity with sleep in under 5s: A systematic review and meta-analysis. Sleep Med Rev. 2020;49:101226.
24. Taylor RW, Gray AR, Heath AM, Galland BC, Lawrence J, Sayers R, et al. Sleep, nutrition, and physical activity interventions to prevent obesity in infancy: follow-up of the Prevention of Overweight in Infancy (POI) randomized controlled trial at ages 3.5 and 5 y. Am J Clin Nutr. 2018;108(2):228-36.
25. Chia MY, L.Y. T, Chua TB. Quality of life and meeting 24-h WHO guidelines among preschool children in Singapore. Early Childhood Education Journal. 2020;48(3):313-23.
26. Kuzik N, Naylor PJ, Spence JC, Carson V. Movement behaviours and physical, cognitive, and social-emotional development in preschool-aged children: Cross-sectional associations using compositional analyses. PLoS One. 2020;15(8):e0237945.
27. Li C, Cheng G, Sha T, Cheng W, Yan Y. The Relationships between Screen Use and Health Indicators among Infants, Toddlers, and Preschoolers: A Meta-Analysis and Systematic Review. Int J Environ Res Public Health. 2020;17(19).
28. Paulus FW, Mohler E, Recktenwald F, Albert A, Mall V. Electronic Media and Early Childhood: A Review. Klin Padiatr. 2021;233(4):157-72.
29. Simonato I, Janosz M, Archambault I, Pagani LS. Prospective associations between toddler televiewing and subsequent lifestyle habits in adolescence. Prev Med. 2018;110:24-30.
30. Aishworiya R, Cai S, Chen HY, Phua DY, Broekman BFP, Daniel LM, et al. Television viewing and child cognition in a longitudinal birth cohort in Singapore: the role of maternal factors. BMC Pediatr. 2019;19(1):286.
31. Carson V, Lee EY, Hesketh KD, Hunter S, Kuzik N, Predy M, et al. Physical activity and sedentary behavior across three time-points and associations with social skills in early childhood. BMC Public Health. 2019;19(1):27.

32. Padmapriya N, Aris IM, Tint MT, Loy SL, Cai S, Tan KH, et al. Sex-specific longitudinal associations of screen viewing time in children at 2-3 years with adiposity at 3-5 years. Int J Obes (Lond). 2019;43(7):1334-43.
33. Chen B, Bernard JY, Padmapriya N, Ning Y, Cai S, Lanca C, et al. Associations between early-life screen viewing and 24 hour movement behaviours: findings from a longitudinal birth cohort study. Lancet Child Adolesc Health. 2020;4(3):201-9.
34. Padmapriya N, Tint MT, Sadananthan SA, Michael N, Chen B, Cai S, et al. The longitudinal association between early-life screen viewing and abdominal adiposity-findings from a multiethnic birth cohort study. Int J Obes (Lond). 2021;45(9):1995-2005.
35. Deng X, He M, He D, Zhu Y, Zhang Z, Niu W. Sleep duration and obesity in children and adolescents: evidence from an updated and dose-response meta-analysis. Sleep Med. 2021;78:169-81.
36. Covington L, Armstrong B, Trude ACB, Black MM. Longitudinal Associations Among Diet Quality, Physical Activity and Sleep Onset Consistency With Body Mass Index z-Score Among Toddlers in Low-income Families. Ann Behav Med. 2021;55(7):653-64.
37. Sparano S, Lauria F, Ahrens W, Fraterman A, Thumann B, Iacoviello L, et al. Sleep duration and blood pressure in children: Analysis of the pan-European IDEFICS cohort. J Clin Hypertens (Greenwich). 2019;21(5):572-8.
38. Cook F, Conway LJ, Giallo R, Gartland D, Sciberras E, Brown S. Infant sleep and child mental health: a longitudinal investigation. Arch Dis Child. 2020;105(7):655-60.
39. Sivertsen B, Harvey AG, Reichborn-Kjennerud T, Ystrom E, Hysing M. Sleep problems and depressive symptoms in toddlers and 8-year-old children: A longitudinal study. J Sleep Res. 2021;30(1):e13150.
40. Reynaud E, Forhan A, Heude B, Charles MA, Plancoulaine S. Night-sleep Duration Trajectories and Behavior in Preschoolers: Results from a Prospective Birth Cohort Study. Behav Sleep Med. 2021;19(4):445-57.
41. Quah PL, Kleijweg J, Chang YY, Toh JY, Lim HX, Sugianto R, et al. Association of sugar-sweetened beverage intake at 18 months and 5 years of age with adiposity outcomes at 6 years of age: the Singapore GUSTO mother-offspring cohort. Br J Nutr. 2019;122(11):1303-12.
42. Vandyousefi S, Davis JN, Gunderson EP. Association of infant diet with subsequent obesity at 2-5 years among children exposed to gestational diabetes: the SWIFT study. Diabetologia. 2021;64(5):1121-32.
