# Supplementary material for: Asia–Pacific consensus statement on integrated 24-hour activity guidelines for the early years
Source: Lancet Reg Health West Pac. 2022 Nov 23;32:100641. doi: 10.1016/j.lanwpc.2022.100641 (PMC9918766; doi:10.1016/j.lanwpc.2022.100641)
Supplement: Supplementary File S2 [file mmc2.docx]

**Asia-Pacific 24-Hour Activity Guidelines for the Early Years Committee**

| **Country** | **First name** | **Last name** |
| --- | --- | --- |
| Australia | Anthony | OKELY |
| Bangladesh | Mohammad Sorowar | HOSSAIN |
| China | Yanjun | ZHAO |
| Fiji | Gade | WAQA |
| Hawaii | Rachel | NOVOTNY |
| Hong Kong | Betty Wai Man | BUT |
| India | Ranjan Kumar | PEJAVER |
| Indonesia | Aman Bakhti | PULUNGAN |
| Japan | Satoshi | KUSUDA |
|  | Ichiro | MORIOKA |
|  | Nobuhiko | NAGANO |
| Malaysia | Thiyagar | A/L NADARAJAW |
|  | Muhammad Yazid | JALALUDIN |
| Mongolia | Ankhmaa | BYAMBAA |
| Myanmar | Mya Sandar | THEIN |
| New Zealand | Rachael | TAYLOR |
| Philippines | Divina Cristy | REDONDO-SAMIN |
| Singapore | Benny Kai Guo | LOO |
|  | Kok Hian | TAN |
|  | Falk | MÜLLER-RIEMENSCHNEIDER |
|  | Benedict | TAN |
|  | Ethel Jie Kai | LIM |
|  | Mohammad | ASHIK |
|  | Ngiap Chuan | TAN |
|  | Ratnaporn | SIRIAMORNSARP |
|  | Oon Hoe | TEOH |
|  | Moira Suyin | CHIA |
|  | Nur Adila | AHMAD HATIB |
|  | Teresa | TAN |
|  | Le Ye | LEE |
|  | Victor Samuel | RAJADURAI |
|  | Phaik Ling | QUAH |
|  | Miriam | LEE |
|  | Aaron | SIM |
| South Korea | Justin | JEON |
| Sri Lanka | Sachith | METTANANDA |
|  | Pujitha | WICKRAMASINGHE |
| Thailand | Areekul | AMORNSRIWATANAKUL |
|  | Pongsak | NOIPAYAK |
| Vietnam | Manh Nhi | HUYNH |
